# ^19^F Electron-Nuclear Double Resonance Reveals Interaction between Redox-Active Tyrosines across the α/β Interface of *E. coli* Ribonucleotide Reductase

**DOI:** 10.1021/jacs.2c02906

**Published:** 2022-06-02

**Authors:** Andreas Meyer, Annemarie Kehl, Chang Cui, Fehmke A. K. Reichardt, Fabian Hecker, Lisa-Marie Funk, Manas K. Ghosh, Kuan-Ting Pan, Henning Urlaub, Kai Tittmann, JoAnne Stubbe, Marina Bennati

**Affiliations:** † Research group ESR spectroscopy, Max Planck Institute for Multidisciplinary Sciences, 37077 Göttingen, Germany; ‡ Department of Chemistry and Chemical Biology, Harvard University, Cambridge, Massachusetts 02138, United States; § Department of structural dynamics, Max Planck Institute for Multidisciplinary Sciences, 37077 Göttingen, Germany; ∥ Department of Molecular Enzymology, Georg-August University, 37077 Göttingen, Germany; ⊥ Research group bioanalytical mass spectrometry, Max Planck Institute for Multidisciplinary Sciences, 37077 Göttingen, Germany; # Bioanalytics, University Medical Center, 37075 Göttingen, Germany; ∇ Department of Chemistry and Department of Biology, Massachusetts Institute of Technology, Cambridge, Massachusetts 20139, United States; ○ Department of Chemistry, Georg-August University, 37077 Göttingen, Germany

## Abstract

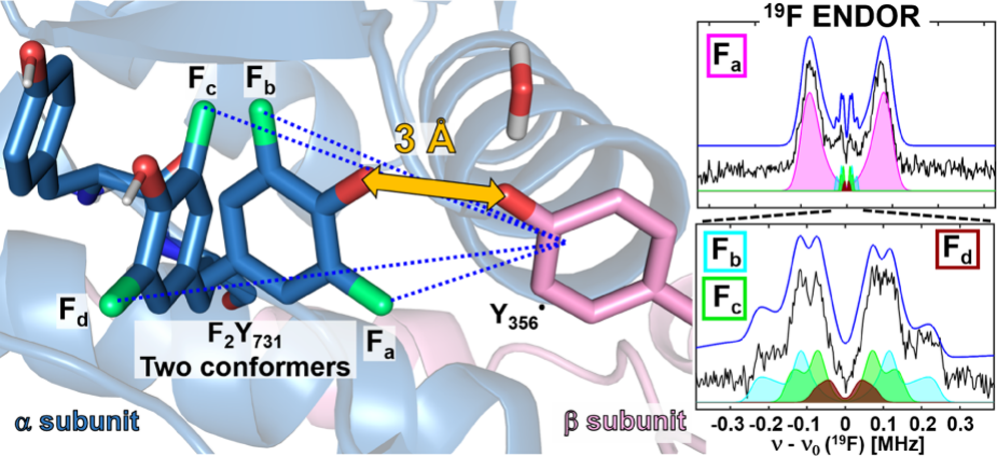

Ribonucleotide reductases (RNRs) catalyze the reduction of ribonucleotides to deoxyribonucleotides, thereby playing a key role in DNA replication and repair. *Escherichia coli* class Ia RNR is an α_2_β_2_ enzyme complex that uses a reversible multistep radical transfer (RT) over 32 Å across its two subunits, α and β, to initiate, using its metallo-cofactor in β_2_, nucleotide reduction in α_2_. Each step is proposed to involve a distinct proton-coupled electron-transfer (PCET) process. An unresolved step is the RT involving Y_356_(β) and Y_731_(α) across the α/β interface. Using 2,3,5-F_3_Y_122_-β_2_ with 3,5-F_2_Y_731_-α_2_, GDP (substrate) and TTP (allosteric effector), a Y_356_^•^ intermediate was trapped and its identity was verified by 263 GHz electron paramagnetic resonance (EPR) and 34 GHz pulse electron–electron double resonance spectroscopies. 94 GHz ^19^F electron-nuclear double resonance spectroscopy allowed measuring the interspin distances between Y_356_^•^ and the ^19^F nuclei of 3,5-F_2_Y_731_ in this RNR mutant. Similar experiments with the double mutant E_52_Q/F_3_Y_122_-β_2_ were carried out for comparison to the recently published cryo-EM structure of a holo RNR complex. For both mutant combinations, the distance measurements reveal two conformations of 3,5-F_2_Y_731_. Remarkably, one conformation is consistent with 3,5-F_2_Y_731_ within the H-bond distance to Y_356_^•^, whereas the second one is consistent with the conformation observed in the cryo-EM structure. The observations unexpectedly suggest the possibility of a colinear PCET, in which electron and proton are transferred from the same donor to the same acceptor between Y_356_ and Y_731_. The results highlight the important role of state-of-the-art EPR spectroscopy to decipher this mechanism.

## 1 Introduction

**Figure 1:**
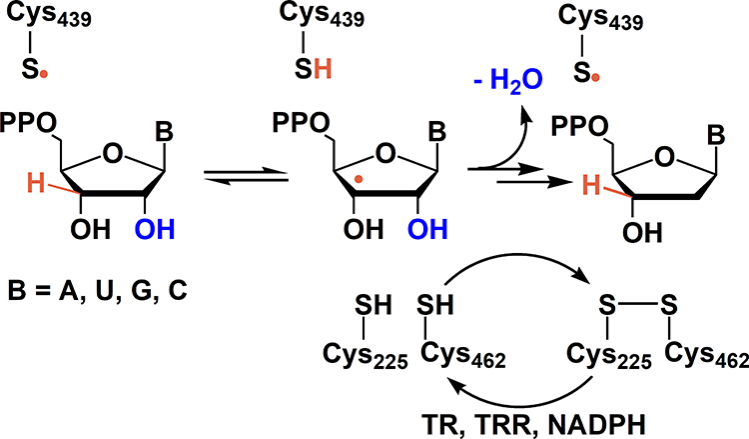
Reduction of NDPs to dNDPs catalyzed by *Escherichia coli* class Ia RNR. The reduction is initiated by a thiyl radical (C_439_^•^), and the reducing equivalents are provided by the oxidation of C_225_ and C_462_ to a disulfide. Multiple turnovers require a redoxin reducing system such as thioredoxin (TR), thioredoxin reductase (TRR), and nicotinamide adenine dinucleotide phosphate (NADPH).

Ribonucleotide reductases (RNRs) catalyze the conversion of four nucleoside di- or triphosphates (ND(T)Ps) to deoxyribonucleoside di- or triphosphates (dND(T)Ps) in all organisms (Figure 1).^1−3^ RNRs are highly regulated enzymes playing an important role in controlling the ratio and relative amounts of dNTPs essential for the fidelity of DNA replication and repair. Imbalance in dNTP pools results in genomic instability and leads to disease states.^4−6^ RNRs’ essential role has made them targets for cancer and, more recently, antibiotic therapeutics.^6−12^

**Figure 2:**
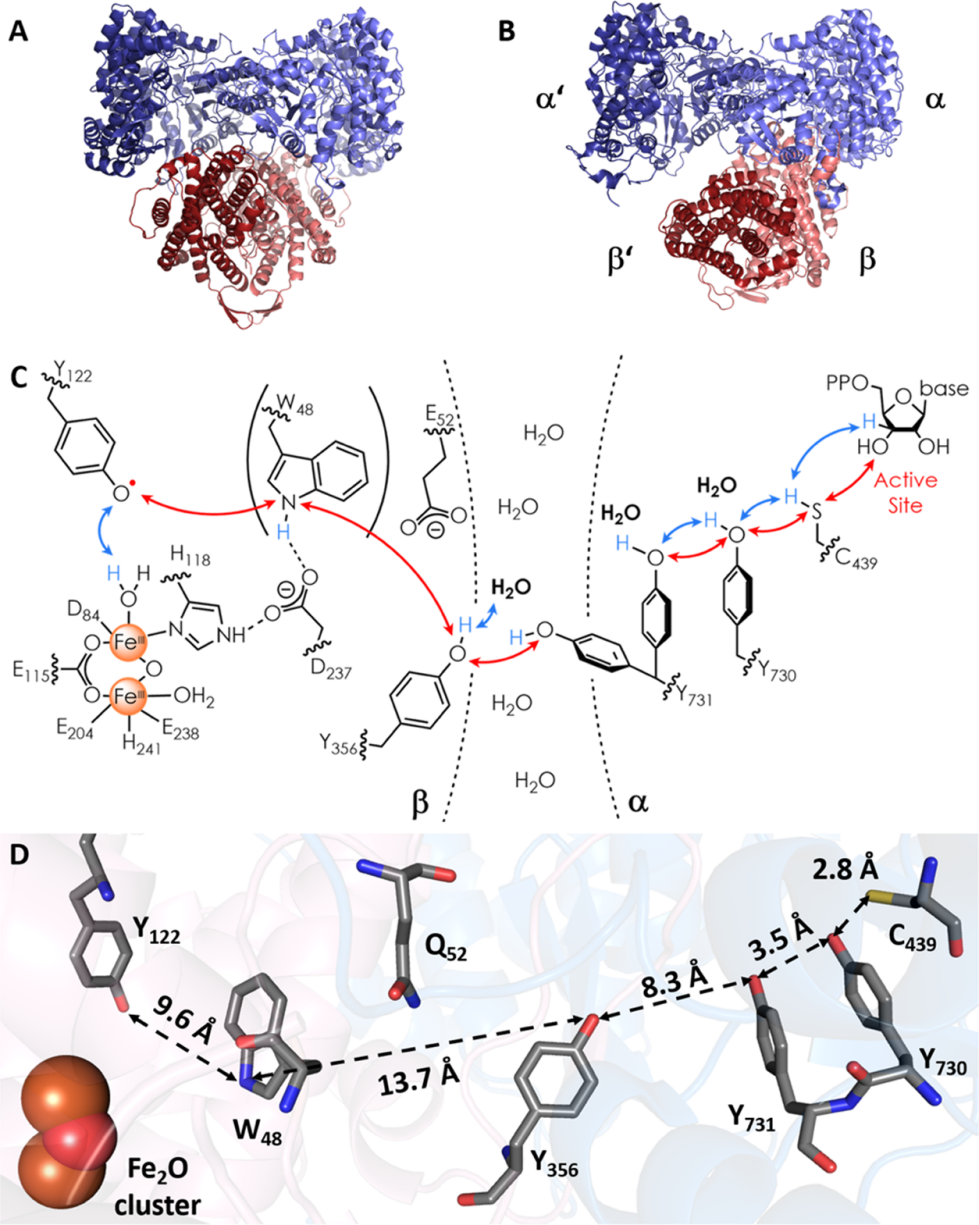
Docking model^13^ (A) and cryo-EM structure^38^ (B) of the α_2_β_2_ complex of *E. coli* class Ia RNR and the proposed RT pathway, (C) and (D), respectively. (A) The docking model based on the shape complementarity of subunits α_2_^13^ and β_2_.^14^ (B) Cryo-EM structure of an α_2_β_2_ complex of RNR generated when E_52_Q/F_3_Y_122_-β_2_, *wt*-α_2_ GDP (substrate) and TTP (effector) were quenched at 50 s (pdb code: 6W4X).^38^ Asymmetry of the complex is indicated by α′β′ (disordered pair) and αβ (ordered pair). (C) The proposed forward RT pathway based on many experiments.^20−27,30−33^ W_48_ is shown in parentheses as there currently is no direct evidence for its involvement. The red and blue double arrows describe electron and proton transfers, respectively. Evidence for the bold water molecules has been reported recently.^27,28^ (D) An intact RT pathway within αβ including Y_356_ and its position relative to Y_731_ is visible for the first time in the cryo-EM structure.^38^ Distances between RT residues are indicated; the ^19^F atoms of 2,3,5-F_3_Y_122_ present in the cryo-EM structure have been omitted. Interfacial residue Q_52_ (E_52_ in *wt*-RNR) is included as it was important for stabilizing the α_2_β_2_ complex in the cryo-EM experiment.

The *E. coli* class Ia RNR, a prototype model system for human RNR,^6^ is composed of two subunits, α^13^ and β,^14^ both required for activity. Based on their α_2_ and β_2_ structures, Uhlin and Eklund proposed a symmetrical α_2_β_2_ docking model (Figure 2A) for active RNR, which has played a central role in the experimental design.^13^ The model for substrate activation and chemistry requires that the diferric tyrosyl radical (Y_122_^•^) cofactor located in β_2_ oxidizes C_439_ to a thiyl radical in the active site of α_2_, which, in turn, initiates NDP reduction (Figures 1 and 2C). Thiyl radical formation is proposed to occur by a radical transfer (RT) pathway, which involves five or six radical intermediates (Figure 2C),^15^ each generated by proton-coupled electron-transfer (PCET) steps.^16−19^

Central for developing this model has been the ability to replace pathway Ys site-selectively with unnatural amino acids (UAAs) that have allowed the generation and thermodynamic trapping of pathway radical intermediates. The tyrosyl radicals (Y^•^s) were studied by a suite of multifrequency electron paramagnetic resonance (EPR)^20−30^ methods as well as by transient absorption spectroscopic methods using photo-β_2_ RNRs.^30−34^

Despite much insight into nature’s design for radical initiation in RNRs, elucidating the molecular basis for the RT across the α/β subunit interface has been hampered by the lack of structural information about the C-terminal tail of all βs (residues 341–375 in *E. coli* RNR), essential for α/β subunit interaction.^35−37^ The location of Y_356_ in the RT pathway within this tail was thus unknown. Recently, a near-atomic resolution cryo-EM structure of a trapped α_2_β_2_*E. coli* complex was obtained (Figure 2B).^38^ It was generated from the incubation of a double mutant of β_2_, E_52_Q/F_3_Y_122_-β_2_, with *wt*-α_2_, substrate (GDP), and allosteric effector (TTP) with freeze-quenching at 50 s. The 2,3,5-F_3_Y_122_ substitution allowed the generation of one dGDP product and accumulation of one pathway radical at Y_356_^•^. The E_52_Q mutation was important for successfully trapping the α_2_β_2_ complex. The E_52_ residue resides at the α/β-interface and is essential for activity, enabling proton release during Y_356_ oxidation in the RT.^33,39^

The cryo-EM structure (Figure 2B) revealed an asymmetric α_2_β_2_ complex, consistent with earlier results.^37,40^ It also revealed the residues in the C-terminal tail of β (341–375) in an ordered αβ pair, the intact RT pathway including the location of Y_356_ and its location relative to Y_731_(α) (Figure 2D) for the first time. The entire C-terminal tail in α′/β′, where chemistry has occurred and Y_356_^•^ is supposedly trapped, remains disordered.

The importance of Y_356_ during RT has been established by many different methods that often led to the detection of the Y_356_^•^ intermediate. Recent studies to identify the proton acceptor during its oxidation in forward RT revealed that the most reasonable candidates, E_52_(β) and E_350_(β), both conserved and essential,^36,39,41^ are unlikely to be the ultimate acceptors.^33,34,42^ These residues are located at ∼7 Å (E_52_) and ∼14 Å (E_350_) distances from the phenol-oxygen atom of Y_356_ in the ordered αβ pair of the cryo-EM structure,^38^ too far for direct proton or H atom transfer with Y_356_.^43^ A variety of ^1^H and ^17^O high-frequency electron-nuclear double resonance (ENDOR) experiments on Y_356_^•^,^27,28^ kinetic studies using RNRs with F*_n_*Y_356_^33^ and a photo-oxidant appended to the C_355_ mutant of β, and pH studies of Y_356_^•^ formation using F_2_Y_356_^42^ all support the interaction of Y_356_^•^ with water (Figure 2C).

Efforts to understand the residues involved in managing the proton to support the PCET between Y_356_ and Y_731_ across the α/β interface have been less successful. The cryo-EM structure shows an O–O distance between Y_356_ and Y_731_ of ∼8 Å in ordered αβ, with Y_731_ in its unusual stacked conformation with Y_730_ as in previous X-ray structures of α_2_ alone.^13^ While a number of pulsed electron double resonance (PELDOR) experiments^6^ revealed sharp distance distributions consistent with little Y_356_^•^ flexibility, several different experiments reported the mobility of Y_731_. In a crystal structure of NH_2_Y_730_-α_2_ alone, Y_731_ was found in a conformation where it is flipped away from the stacked conformation with NH_2_Y_730_.^44^ PELDOR studies on a double mutant R_411_A-NH_2_Y_731_-α_2_ under turnover conditions revealed a conformational change of 3 Å in trapped NH_2_Y_731_^•^, consistent with a flipping toward the α/β interface.^26^ Subsequent studies using photo-β_2_ with the same α_2_ mutations revealed dynamic/rapid conformational changes of Y_731_.^30^ Another EPR study by Yokoyama et al. suggested the flipping of F_2_Y_731_^•^,^23^ which was trapped as a minority radical species in NO_2_Y_122_-β_2_/F_2_Y_731_-α_2_. Molecular dynamics (MD) simulations using the cryo-EM structure and the α/β interface in water also support the flexibility of Y_731_,^45^ with movement away from the stacked conformation with Y_730_. The studies together support a model for PCET between Y_356_^•^ and Y_731_ across the α/β interface that could involve a movement of Y_731_ toward the interface (Figure 2C), with consequences for their PCET chemistry. However, structural or spectroscopic evidence for interaction between Y_356_^•^ and Y_731_ has never been observed.

In this article, we use ^19^F–Y analogues introduced site-specifically into *E. coli* RNR, F_3_Y_122_-β_2_ (or the double mutant E_52_Q/F_3_Y_122_-β_2_), incubated with 3,5-F_2_Y_731_-α_2_, GDP, and TTP to generate and trap Y_356_^•^. F_2_Y_731_ was chosen for its symmetric ^19^F substitution pattern and minimally perturbed reduction potential relative to Y.^46,47^ The Y_356_^•^ location and identity are established using 34 GHz PELDOR and 263 GHz EPR spectroscopies, respectively. ^19^F ENDOR spectroscopy^48,49^ at 94 GHz is used in an effort to determine the distances across the subunit interface between the trapped Y_356_^•^(β) and the ^19^F nuclei of F_2_Y_731_(α). The ENDOR spectra give unambiguous evidence for two conformations of F_2_Y_731_. One conformation is consistent with the structure observed by cryo-EM (ordered αβ pair). The second conformation indicates a flipping of F_2_Y_731_ toward Y_356_^•^. The results have important implications for the PCET mechanism across the α/β interface.

## 2 Materials and Methods

### 2.1 Preparation of RNR Mutants and Activity Assays

The RNR mutants F_3_Y_122_-β_2_, E_52_Q/F_3_Y_122_-β_2_, F_2_Y_731_-α_2_, and ^17^O–Y-*wt*-α_2_ were expressed and purified, as previously described.^39,44,50^ Activities of (E_52_Q)F_3_Y_122_-β_2_/F_2_Y_731_-α_2_ and *wt*-β_2_/^17^O–Y-α_2_ were determined using the spectrophotometric assay (Supporting Information (SI) 1, Table S1).^51^

### 2.2 EPR Sample Preparation

The Y_356_^•^ intermediate was trapped by incubating a solution of F_2_Y_731_-α_2_, GDP, and TTP in assay buffer (50 mM HEPES, 15 mM MgSO_4_, 1 mM EDTA, pH 7.6) with F_3_Y_122_-β_2_ or E_52_Q/F_3_Y_122_-β_2_ in assay buffer. Glycerol concentrations were optimized (Figure S1) and typically added to ∼20% of the final volume to prolong phase memory times *T*_M_ for PELDOR and ENDOR measurements. The final concentrations were ∼80 μM α_2_β_2_, ∼1 mM GDP, and ∼200 μM TTP. The reaction mixture was transferred to either 34 GHz EPR tubes (Q-band) (12 μL, 1.5 mm inner diameter (ID) Suprasil tube, Wilmad) or 94 GHz (W-band) tubes (4.4 μL, 0.7 mm ID clear fused quartz tubes) and quenched by freezing in liquid nitrogen at reaction times (*T*_Q_) of 40–80 s (Q-band) or 35–55 s (W-band). A second set of samples were prepared with *T*_Q_ > 100 s. Two hundred and sixty-three GHz EPR samples were prepared in Suprasil capillaries (ID 0.2 mm, Vitrocom) without glycerol and quenched at *T*_Q_ = 15–20 s. All samples are summarized in SI 2, Table S2.

### 2.3 263 GHz EPR Spectroscopy

High-frequency (HF) 263 GHz echo-detected EPR spectra were recorded with a commercial spectrometer, as previously reported.^52^ Details on the spectral acquisition are given in SI 3.

### 2.4 34 GHz PELDOR Spectroscopy

Four-pulse PELDOR experiments^53,54^ were performed at 34 GHz (Q-band) on a commercial Bruker ELEXSYS E580 EPR spectrometer, as previously reported.^27^ An optimized temperature of 50 K was selected, where high sensitivity is achieved and unreacted F_3_Y_122_^•^ does not contribute to the spin echo under conditions used for data collection (SI 4.1–4.3). MW pulses were amplified by a pulsed 170 W TWT amplifier (Model 187Ka, Applied Systems Engineering) with typical pulse lengths of 14–16 ns for the pump π-pulse at the center of the overcoupled resonator. The observer frequency was set to −105 MHz from the dip center, leading to observer π-pulse lengths of 24–28 ns. The *τ*_1_ value was 250 ns, and τ_2_ values were optimized based on *T*_M_ measurements (SI 4.2). Shot repetition times were 4–6 ms. Time traces were recorded at three different observer positions (Figure S5) and their intensities were summed, reflecting their respective EPR signal strengths at that excitation position. Traces were analyzed with DeerAnalysis 2019,^55^ using Tikhonov regularization (*L*-curve criterion for α parameter) and checked for consistency using neural network analysis.^56,57^

### 2.5 94 GHz ENDOR Spectroscopy

Pulsed EPR and ENDOR experiments at 94 GHz (W-band) were performed on a commercial Bruker ELEXSYS E680 EPR spectrometer, as previously described.^25^ Using a 2 W MW amplifier, typical π/2 pulse lengths of 10–12 ns were achieved. EPR (echo-detected) spectra and signal contributions are illustrated in SI 5.1. Shot repetition times were optimized to 2–4 ms based on *T*_1_ measurements (SI 5.2).

^19^F Mims ENDOR spectra of the Y_356_^•^ were recorded using radio frequency (RF) pulses amplified by a 250 W RF amplifier (250A250A Amplifier Research). RF pulse lengths of 22 μs were used for ^19^F nuclei with ∼1.6 MHz couplings or 44 μs for couplings ≤∼250 kHz. RF pulse lengths were optimized using Rabi nutation experiments. Stochastic RF acquisition^58−60^ with 20 shots per point was used. To observe ^19^F couplings of different sizes, the adjustment of the interpulse delay τ in the Mims sequence was crucial. For couplings on the order of 1.6 MHz, two measurements with τ values of 236 and 266 ns were performed and summed subsequently (normalized to the number of scans) to attenuate the proton background. For smaller couplings, ≤∼250 kHz, τ was optimized to 620–622 ns (SI 5.3). ENDOR spectra were recorded at three different observer positions (Figure S8) and summed up with intensities reflecting their respective EPR signal strengths at that excitation position.

Data were collected at two temperatures. At 50 K, ENDOR sensitivity was higher than that at 80 K, where usually the signal of unreacted F_3_Y_122_^•^ disappears due to faster relaxation.^27^ As a downside, at 50 K, the unreacted F_3_Y_122_^•^ contributed to the echo intensity of the Mims sequence at short interpulse delays τ. The contribution of F_3_Y_122_^•^ led to ^19^F ENDOR background signals, which had to be removed during data processing (SI 5.4). As a control for the background correction procedure, we repeated representative ^19^F ENDOR measurements at 80 K (SI 5.5–5.6) where no background of F_3_Y_122_^•^ was present. The results obtained at 50 and 80 K are fully consistent. In addition to the ^19^F background, broad, overlapping ^1^H resonances associated with the 3,5-H atoms of Y_356_^27^ were identified by their changes observed with τ value changes and they were subtracted from the ^19^F spectra, as illustrated in SI 5.4.

^17^O ENDOR control experiments were performed using similar parameters described in our recent ^17^O ENDOR study^28^ and are reported in SI 6.

### 2.6 Simulations of ENDOR Data

Mims ENDOR simulations of the Y_356_^•^ were performed using EasySpin’s saffron routine.^61^ The *g* tensor was *g*_*x*_ = 2.0062, *g*_*y*_ = 2.0044, and *g*_*z*_ = 2.0022.^27^ In the molecular frame, *g*_*x*_ is aligned along the C–O^•^ bond of Y_356_^•^, while *g*_*y*_ is perpendicular to this direction and in the plane of the aromatic ring. The strongly coupled β-proton of Y_356_^•^ was included using previously reported hyperfine coupling (HFC) parameters.^27^ For simulating the ^19^F ENDOR spectra with τ = 620–622 ns, the C3 and C5 protons^27^ of Y_356_^•^ were included. The ^19^F ENDOR line width parameter was simulated as 25 kHz for couplings below 0.5 MHz.^49^ For larger couplings, a line width of 250 kHz was used. Chemical shift anisotropies were not resolved in the 94 GHz ^19^F ENDOR spectra.^62^

### 2.7 Structural Models for ENDOR Analysis

**Figure 3:**
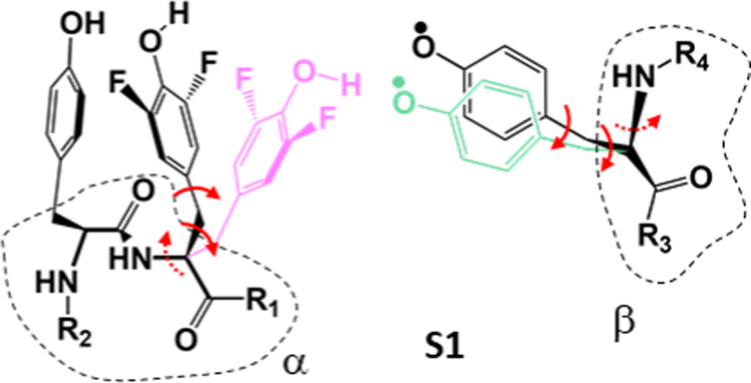
Models for the Y-triad. The black conformation corresponds to **S1** but without the water molecule. The pink orientation of F_2_Y_731_ illustrates a flipped conformation, and the green orientation of Y_356_^•^ represents a repositioning of the radical toward F_2_Y_731_, used in models **S2**–**S5**. Atom positions of the backbone are from the cryo-EM structure within ≤∼0.5 Å. R_1_–R_4_ peptide chains have been replaced by H atoms in **S1**–**S5**. Red arrows indicate a rotation around a bond, and dashed arrows indicate small rotations (Table S6).

Due to the large parameter space associated with the two Fs of F_2_Y_731_ and, as will become clear, their multiple side-chain conformations, a fitting routine that generates the most likely set of HFC parameters by minimizing residuals (rmsd) is not possible. We therefore used an approach similar to that described previously to analyze the PCET steps within α_2_ using NH_2_Y_731_ and the X-ray structure of α_2_ to position Y_730_ and C_439_.^25^ In the present case, the small models were constructed starting from pdb 6W4X, the recent cryo-EM structure (resolution 3.3–5.5 Å).^38^ Y_356_ from β and Y_731_ and Y_730_ from α were extracted from the ordered α/β pair (Figure 2B,D). ^19^F atoms at C3 and C5 of Y_731_ were introduced using PyMOL.^63^ The peptide bonds connecting each tyrosine to their protein backbone were replaced by NHR and −CRO (Figure 3) groups, and their *xyz* coordinates were not changed compared to the cryo-EM structure. Density functional theory (DFT)-based, constrained geometry optimization using ORCA^64^ resulted in the model structure **S1** of the triad Y_356_–F_2_Y_731_–Y_730_. Further representative conformations of the triad were obtained by rotating around Cα/Cβ and Cβ-phenol bonds displacing the phenol side chains of Y_356_ and F_2_Y_731_, as illustrated in Figure 3. Resulting models to fit the spectroscopic data are designated **SX** (X =1, 2, 3,...5) and are summarized in Tables S6 and S7 in SI 8. A water molecule binding to Y_356_^•^ was also introduced into each model, with a binding geometry based on our previous studies (H-bond length ca. 1.8 Å, angle C4–O^•^···H ca. 120°, C3–C4–O^•^···H dihedral ca. 20°).^27,28^ The effect of H-bonds on the spin density distribution,^65,66^ further technical details on the DFT calculations, and the adaptation of the DFT-predicted parameters to the ENDOR simulations are described in the results section and summarized in SI 7. Contributions of the different conformations were assessed by rmsd analysis. Orientation-selective ^19^F spectra were then simulated using one set of parameters for all spectra.

## 3 Results

### 3.1 Characterization of RNR Constructs Using Activity Measurements, High-Field EPR, and PELDOR

The first part of the investigation required examination of the new RNR constructs that contain the ^19^F labels in F_2_Y_731_. Steady-state activities are reported in Table S1. Spectrophotometric assays revealed a specific activity of 560 nmol/(mg·min) (ca. 7% of wt) for F_3_Y_122_-β_2_/F_2_Y_731_-α_2_, defined with respect to the mass of β_2_ in the assay. In contrast, an activity of only 6 nmol/(mg·min), that is, the lower limit of detection, was measured for E_52_Q/F_3_Y_122_-β_2_/F_2_Y_731_-α_2_. The latter finding was expected, as the E_52_Q mutation disrupts steady-state activity.^39^

Nevertheless, both constructs are capable of one turnover and allowed trapping of the intermediate Y_356_^•^ for EPR samples during back-radical transfer.^67^ Moreover, glycerol is required in the sample preparation to prolong spin relaxation in the EPR experiments. Thus, the glycerol content (v%) was also optimized based on its effect on RNR activity (SI 1) and a value of 20 v% was selected for almost all samples (SI 2, Table S2). We characterized the structure of the trapped radical in F_3_Y_122_-β_2_/F_2_Y_731_-α_2_ and E_52_Q/F_3_Y_122_-β_2_/F_2_Y_731_-α_2_ by 263 GHz EPR (SI 3). In all quenched reaction mixtures, two radical species were observed (Figure S2). One contribution arose from the unreacted F_3_Y_122_^•^ and was readily identified by its large *g*_*x*_ value (2.0082) and its characteristic ^19^F HFC structure. After subtracting a reference spectrum of F_3_Y_122_^•^, the spectrum of the intermediate became visible (Figure S3). This radical was identified as Y_356_^•^ due to the characteristic low *g*_*x*_ value of 2.0062 (reference spectrum of Y_356_^•^ is shown in Figures S2 and S3), as reported with F_3_Y_122_-β_2_/*wt*-α_2_.^27^ The analysis of the HF-EPR spectra also revealed no other radical species.

**Figure 4:**
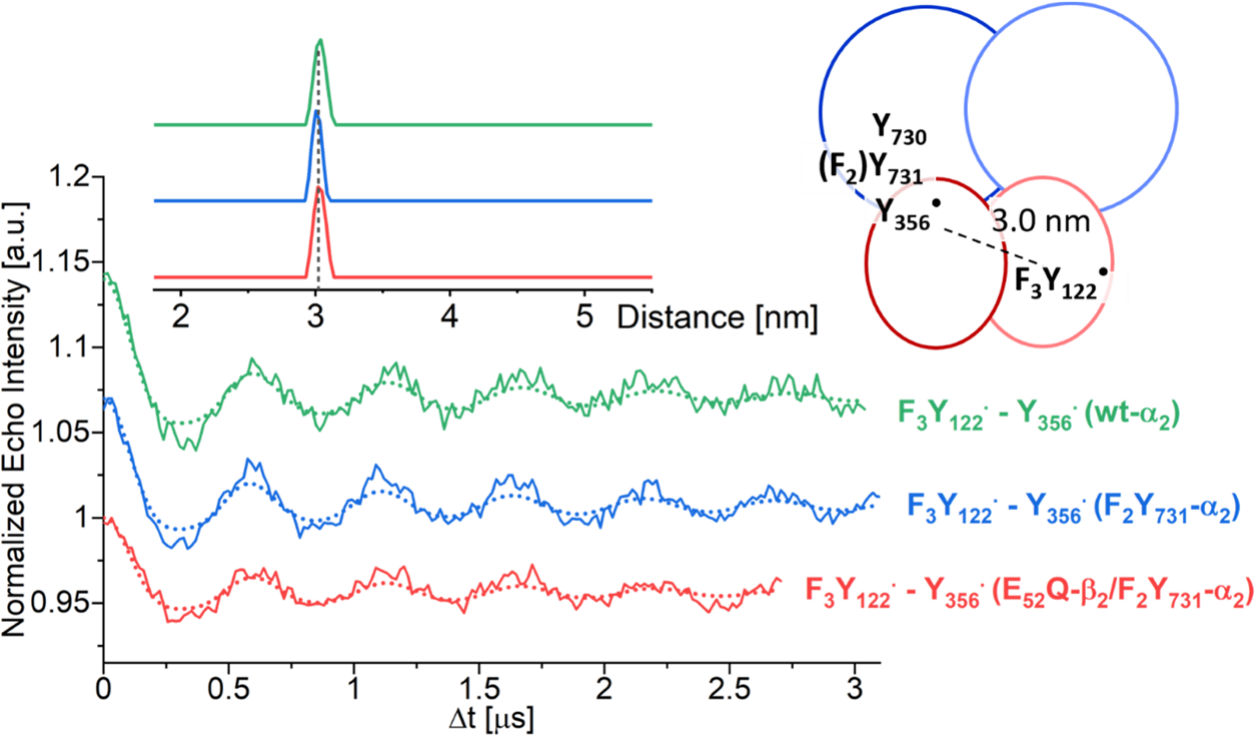
Orientation-averaged 34 GHz PELDOR time traces of F_3_Y_122_-β_2_/F_2_Y_731_-α_2_ (∼80 μM, *T*_Q_ = 77 s, blue line), E_52_Q/F_3_Y_122_-β_2_/F_2_Y_731_-α_2_ (∼80 μM, *T*_Q_ = 44 s, red), and F_3_Y_122_-β_2_/*wt*-α_2_ (green) along with fits (dotted lines). Distance distributions are shown as the inset. A cartoon illustrates the assignment of distance peaks to radical pairs. A symmetric representation was chosen as the experiments reported herein do not inform about the asymmetry in the protein complex.

PELDOR spectroscopy (34 GHz) was then used to measure the diagonal distance between Y_356_^•^ in one αβ pair and F_3_Y_122_^•^ in the second one (Figure 4). The orientation-averaged time traces exhibit clear oscillations. Indistinguishable results were obtained for various sample preparation conditions (SI 4). For comparison, a time trace of F_3_Y_122_-β_2_/*wt*-α_2_ was also measured (Figure 4, green). Distance distributions with a single peak centered at 3.03 ± 0.02 nm (Figure 4) and a width (full width at half-maximum (FWHM); Table S4) of 0.09–0.14 nm were obtained for all samples. The observed distance is typical for F_3_Y_122_^•^–Y_356_^•^ pairs.^6,27^ From PELDOR and HF-EPR, we conclude that Y_356_^•^ is the observed radical, as previously characterized using *wt*-α_2_ for incubation.^27^

It is interesting to consider the observed distance within the framework of the new cryo-EM structure.^38^ The detected radical intermediate (Y_356_^•^) is thought to be produced during reverse RT in the first turnover.^67^ If the first turnover was occurring for instance in the α′β′ pair, see the notation from the cryo-EM structure (Figure 2B), then the observed PELDOR distance should be between Y_356_^•^(β′) and F_3_Y_122_^•^(β). However, in the cryo-EM structure, the C-terminal β′ tail is disordered at the interface, indicating that the trapped state might be different under the conditions of the EPR experiments. Because of the disorder, the distance between F_3_Y_122_^•^(β) and Y_356_^•^(β′) cannot be measured in the cryo-EM structure. If we consider the opposite diagonal distance, i.e., between the centroids^68^ of the Tyr-O, C1, C3, and C5 atoms of F_3_Y_122_^•^ in β′ and Y_356_^•^ in β, then the PELDOR distance of 3.0 nm is in agreement with this structure. We note that many such distances have been measured with other constructs.^6^ All give a sharp 3 nm distance feature, suggesting that the Y_356_^•^ conformation is constrained. Our model for half-site RNR reactivity^15^ requires that the complex interconverts to allow for alternating PCET in αβ and α′β′. When the Y_356_^•^ is trapped, the interconversion is slow. The kinetics of this structural interconversion and the mechanism of switching remain to be established but are likely to be critical for comparing results from different experimental setups.

### 3.2 Distance Measurements across the RNR α/β Interface Using 94 GHz ^19^F ENDOR

#### 3.2.1 ^19^F ENDOR Detects Y_356_^•^–^19^F_2_Y_731_ Distances

**Figure 5:**
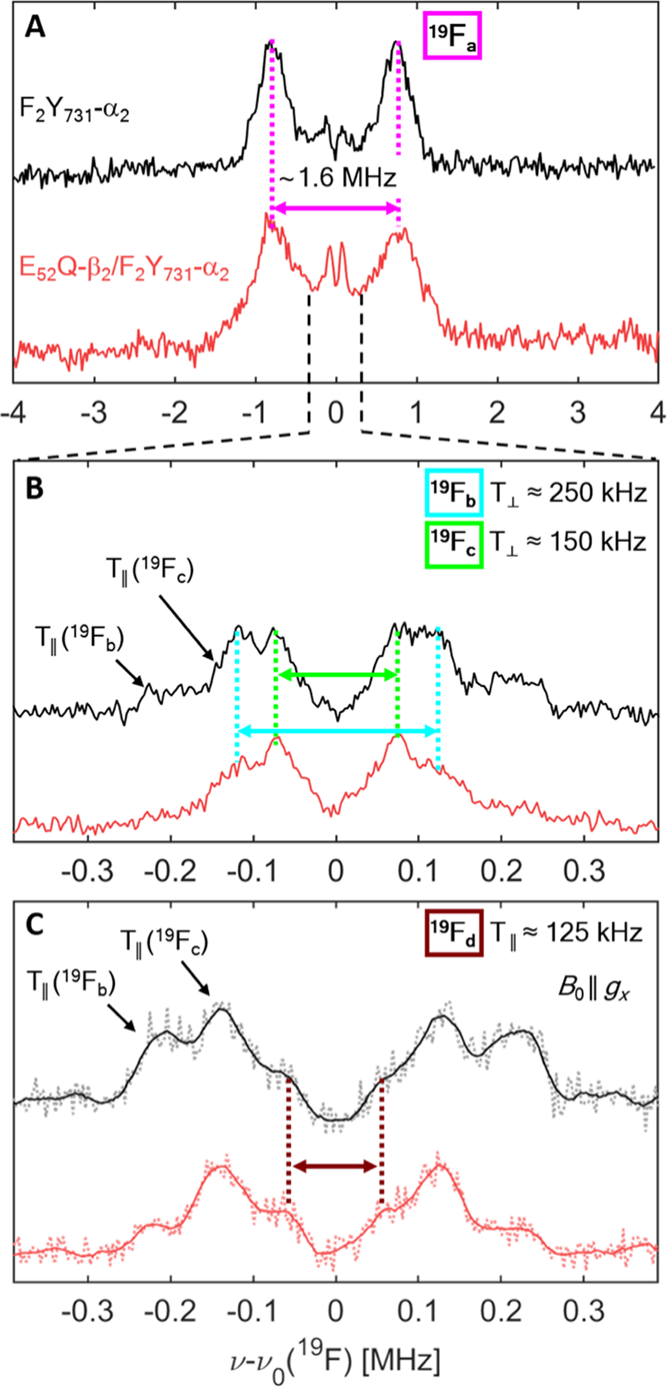
94 GHz ^19^F Mims ENDOR spectra of F_3_Y_122_-β_2_/F_2_Y_731_-α_2_ (80 μM, *T*_Q_ = 50 s, black lines) and E_52_Q/F_3_Y_122_-β_2_/F_2_Y_731_-α_2_ (80 μM, *T*_Q_ = 35 s, red lines) at *T* = 50 K. Spectra in panels (A) and (B) were obtained by adding three orientation-selective spectra. (A) Measurement with short τ values (∼250 ns). (B) Measurement with larger τ values (∼620 ns). (C) Orientation-selective spectra with *B*_0_ a∥ *g*_*x*_ and τ = 620 ns after data point smoothing with the Savitzky–Golay filter (full lines). Original data are shown as dotted lines. Measurement time per spectrum is 30–40 h (A) and 50–60 h (B). Analysis of the spectra in panels (A)–(C) requires consideration of four nuclei ^19^F_a_–^19^F_d_, as marked by arrows and colored dashed lines.

^19^F ENDOR spectra of Y_356_^•^ in F_3_Y_122_-β_2_/F_2_Y_731_-α_2_ (black) and E_52_Q/F_3_Y_122_-β_2_/F_2_Y_731_-α_2_ (red) were obtained after summing three background-corrected, orientation-selective spectra in the range of ±4 MHz around the ^19^F Larmor frequency ν_0_(^19^F) (Figure 5A). When using short τ values (236 and 266 ns), prominent resonances are observed at ±∼0.8 MHz in both samples. These resonances are attributed to one ^19^F nucleus, F_a_, with a peak separation of ∼1.6 ± 0.1 MHz (purple, dashed lines). Additionally, sharp features are observed in a ±250 kHz region around ν_0_(^19^F). These resonances were investigated using a larger τ value of 620 ns, which enhances the sensitivity for smaller couplings (Figure 5B).^49^ For both samples, the spectra in Figure 5B can be interpreted as a superposition of two Pake patterns contributed by two ^19^F nuclei, designated as F_b_ and *F*_c_. Pake patterns result from purely dipolar coupling and allow assignment of the corresponding dipolar HFC *T* by reading off the splitting between the sharp, central peaks: *T*_b_ = 250 ± 15 kHz (cyan, dashed lines) and *T*_c_ = 150 ± 15 kHz (green, dashed lines). These peaks are contributed by molecules in which the ^19^F-radical interspin vector is perpendicular to the external magnetic field *B*_0_. Using the point-dipole approximation (eq 1)^49^
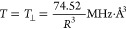
1we can estimate interspin distances of *R*_b_ = 6.7 ± 0.2 Å and *R*_c_ = 7.9 ± 0.3 Å, with the centroid of the O, C1, C3, and C5 atoms of Y_356_^•^ as a point of reference.^68^ Aside from the central peaks, Pake patterns are also characterized by shoulders appearing at twice the coupling strength (2·*T* = *T*_∥_). These features are contributed by molecules with interspin vectors parallel to *B*_0_. The dipolar approximation does not apply for the stronger coupling *T*_a_ due to the shorter distance, <5 Å.^49^

The observation of three distinct ^19^F resonances in Figure 5A,B requires at least two conformations of F_2_Y_731_. Since each conformation contributes two ^19^F–Y_356_^•^ spin pairs, a fourth set of resonances (F_d_) is expected but not clearly resolved in the spectra obtained by summing up three orientation-selective measurements. An indication for coupling to a fourth nucleus F_d_ was provided by the orientation-selective measurements with *B*_0_ aligned along *g*_*x*_ (Figure 5C). Here, strong selectivity for the parallel components of F_b_ and F_c_ was observed. In addition, shoulders on the inside of the two most prominent features are observed, which suggest the parallel coupling of the fourth atom F_d_. Further analysis of the orientation-selective spectra is discussed below and will confirm this assignment.

Interestingly, the size of the observed HFCs (peak positions) is conserved in both F_3_Y_122_-β_2_/F_2_Y_731_-α_2_ and E_52_Q/F_3_Y_122_-β_2_/F_2_Y_731_-α_2_ mutants, but the spectrum of E_52_Q/F_3_Y_122_-β_2_/F_2_Y_731_-α_2_ in Figure 5A appears broader, suggesting more heterogeneity in this mutant.

#### 3.2.2 Examination of Structural Models of the Triad Y_730_–F_2_Y_731_–Y_356_^•^

To rationalize the ^19^F ENDOR spectra, structural models of the tyrosine triad were built (Section 2.7 and Figure 3) and the DFT-predicted ^19^F HFCs were compared with the experimental values in Figure 5. The starting point for modeling is the cryo-EM structure.^38^ Model **S1** (Figure 3, black) is identical to this structure, with two ^19^F nuclei replacing the 3,5-H atoms in Y_731_. This structure results in HFCs of 65 kHz and 114 kHz (see also SI 8, Table S8), the latter approaching but not quite matching the 150 kHz indicated for F_c_ in Figure 5B given DFT uncertainties up to 20%. The 65 kHz coupling could potentially be attributed to the fourth ^19^F nucleus, F_d_.

**Figure 6:**
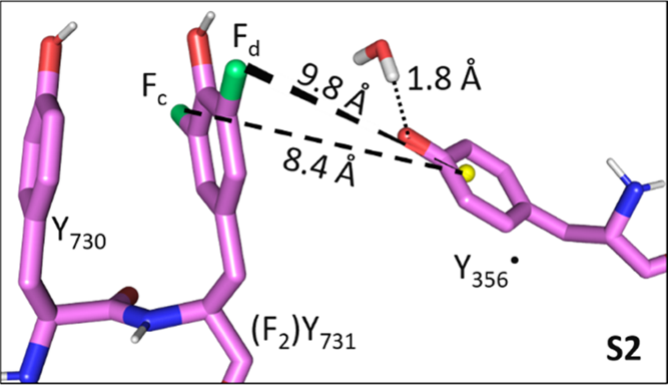
Model **S2**, ^19^F–Y_356_^•^ distances are indicated by dashed lines (centroid of Y_356_^•^ as a point of reference). Fluorine, oxygen, and nitrogen atoms are in green, red, and blue, respectively. H_2_O was included based on our previous results.^27,28^

To increase the coupling strength in **S1**, either the position of F_2_Y_731_ or of Y_356_^•^ had to be readjusted for the spin centers to come closer. An increase of *T*_c_ from 114 to ∼150 kHz for F_c_ would require reducing the interspin distance by roughly 1 Å based on eq 1. To maintain the stacked arrangement of F_2_Y_731_ and Y_730_, observed in almost all available structures, we adjusted the position of O–Y_356_^•^ by ca. 1 Å, which is still well within the resolution of the cryo-EM structure, as indicated in green color in Figure 3 (Table S6). This resulted in model **S2**, illustrated in Figure 6. We note that in model **S2**, as well as in all other models, a water molecule was introduced in the vicinity of Y_356_^•^ (Section 2.7), the presence of which was reported earlier.^27,28^ The H-bonding water molecule affects Y_356_^•^’s spin density distribution and, consequently, also the effective ^19^F-radical HFCs. As detailed in SI 7, the resulting geometrical changes are minor and amount to ca. 0.1–0.2 Å.

In **S2**, the ^19^F–Y_356_^•^ distances are 9.8 and 8.4 Å, the latter consistent with the estimate for *R*_c_ based on the dipolar approximation (eq 1). DFT analysis of **S2** predicts coupling constants of 85 and 153 kHz, reproducing the coupling of F_c_ in Figure 5B within the estimated uncertainty. The 85 kHz coupling could be attributed to F_d_. When the triad shown in **S2** is incorporated back into the cryo-EM structure, the position of Y_356_^•^ was found to fulfill the PELDOR diagonal distance of 3.0 nm (Figure 4 and Table S7).

Nevertheless, it is clear that neither model **S2** nor reorienting the ring plane of F_2_Y_731_ (model **S3**, Figure S16) is able to reproduce the observed strong HFCs of F_a_.

**Figure 7:**
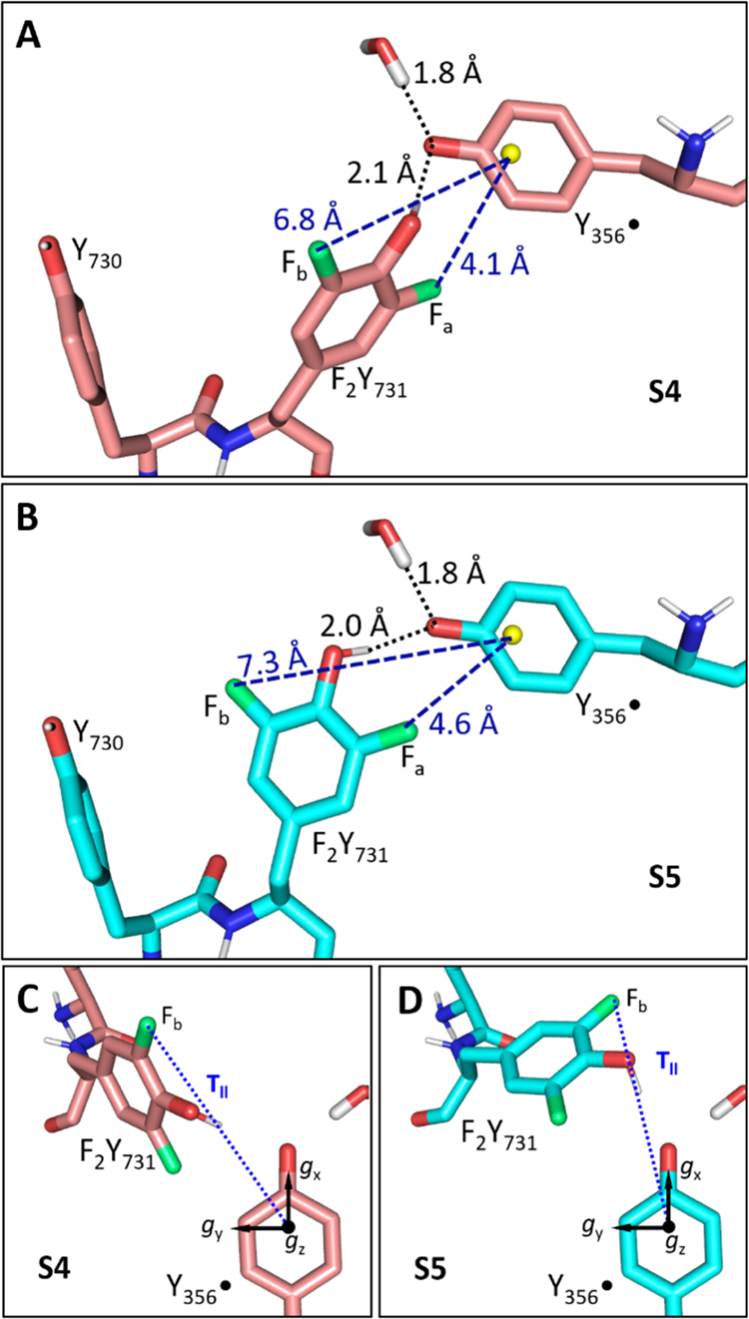
Models **S4** (A) and **S5** (B). (A) Model **S4** (fluorine, oxygen, and nitrogen atoms in green, red, and blue, respectively). H-bond lengths and the ^19^F-centroid (Y_356_^•^, yellow sphere) distances are indicated. (B) Model **S5** (cyan sticks, colors as in panel A). (C and D) Top view of the models shown in panels (A) and (B). In panels (C) and (D), the *g* tensor of Y_356_^•^ is indicated along with the parallel component of the dipolar HFC tensor of the distal ^19^F nucleus F_b_.

We therefore examined the possibility that a second conformation between the interfacial Ys might result in a second pair of stronger ^19^F HFCs. This proposal is reasonable based on previous evidence from different types of experiments that Y_731_ can flip.^23,26,30,44,45^ A small model based on the flipped Y-dyad taken from the X-ray structure of NH_2_Y_730_-α_2_^44^ (without β_2_) could not be placed into the cryo-EM structure using pair fitting (in PyMOL) of the ring atoms to superimpose the Y_730_ side chains since clashes resulted (SI 8, Figure S17). This is in principle expected because this structure is missing the β subunit, which provides structural constraints. We thus focused on αβ and returned to model **S2**, adjusted the dihedral angles around Cα–Cβ and N–Cα of Y_731_ (Table S6), until the DFT-predicted HFC couplings reached the range of the experimental values for F_a_ and F_b_. Representative structures that fulfilled the ^19^F HFCs are shown as models **S4** and **S5** (Figure 7), in which the fluorophenol groups are flipped by about 50–70° toward the subunit interface.

In **S4** (Figure 7A,C), the ^19^F nuclei reside at distances of 4.1 and 6.8 Å from the centroid of Y_356_^•^. For the proximal ^19^F atom (F_a_), DFT predicts a dipolar coupling constant *T*_a_ of ∼1.0 MHz and a negative, isotropic coupling constant *a*_iso,a_ of −0.8 MHz. This combination leads to a splitting of ∼1.8 MHz for **S4**, similar to the ∼1.6 MHz observed experimentally for F_a_ (Figure 5A). The larger of the two ^19^F-radical distances in **S4** agrees well with the estimate for *R*_b_, yielding a coupling constant *T*_b_ of 254 kHz, in agreement with the resonances of F_b_ (Figure 5B).

In a second model with a flipped Y_731_ (**S5**, Figure 7B,D), a distinct orientation of Y_731_ and Y_356_ was considered to account for orientation selection (see also next section). In **S5**, the ^19^F–Y_356_^•^ distances are 4.6 and 7.3 Å. The interspin vector from the distal F_b_ to the centroid of Y_356_^•^ is nearly parallel to the direction of *g*_*x*_ (Figure 7D) and distinct from **S4** (Figure 7C). It has a DFT-derived HFC of *T*_b_ = 246 kHz. For the proximal ^19^F nucleus F_a_, a dipolar coupling constant of *T*_a_ ≈ 0.8 MHz with a negative isotropic coupling constants *a*_iso,a_ of ca −1.0 MHz is predicted and leads to an expected peak separation of ∼1.8 MHz as in **S4**.

**Figure 8:**
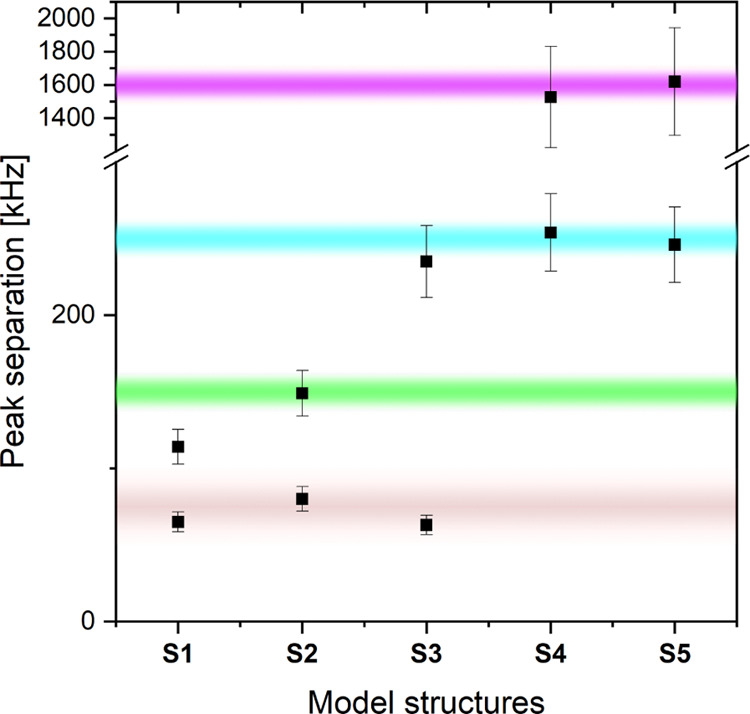
Comparison of experimentally observed peak separation from Figures 5 and 9 (purple (F_a_), cyan (F_b_), green (F_c_), and brown (F_d_) shadings indicate the range of uncertainty) with DFT-predicted peak positions (black squares) for models **S1**–**S5**. For the DFT values, an error of ±20% (F_a_, this nucleus exhibits isotropic and anisotropic coupling) or ±10% (F_b_–F_d_, these nuclei show purely dipolar coupling) is estimated.

A comparison of DFT-predicted HFCs from all models, **S1**–**S5**, and the experimental values is shown in Figure 8. More details on geometrical parameters of the five models are summarized in Table S7. We note that the combination of **S2** with either **S4** or **S5** could satisfy the experimentally observed peak separations in Figure 5.

Finally, both **S4** and **S5**, when integrated back into the framework of the cryo-EM structure,^38^ give centroid–centroid distances between F_2_Y_731_ in αβ and F_3_Y_122_^•^ in α′β′ of 35.0 and 35.5 Å, respectively, both very similar to the constraints measured in our previous PELDOR experiments.^26^

#### 3.2.3 Spectral Simulations Including a Superposition of Stacked and Flipped Y_731_ Conformations

The DFT analysis indicated that it is possible to find mutual conformations of F_2_Y_731_ and Y_356_, which individually satisfy some observed ^19^F–Y_356_^•^ distances. To examine whether a superposition of these conformations can reproduce the ENDOR spectra, we also considered the orientation-selected ENDOR spectra, which pose additional constraints with respect to the sum spectra of Figure 5.

**Figure 9:**
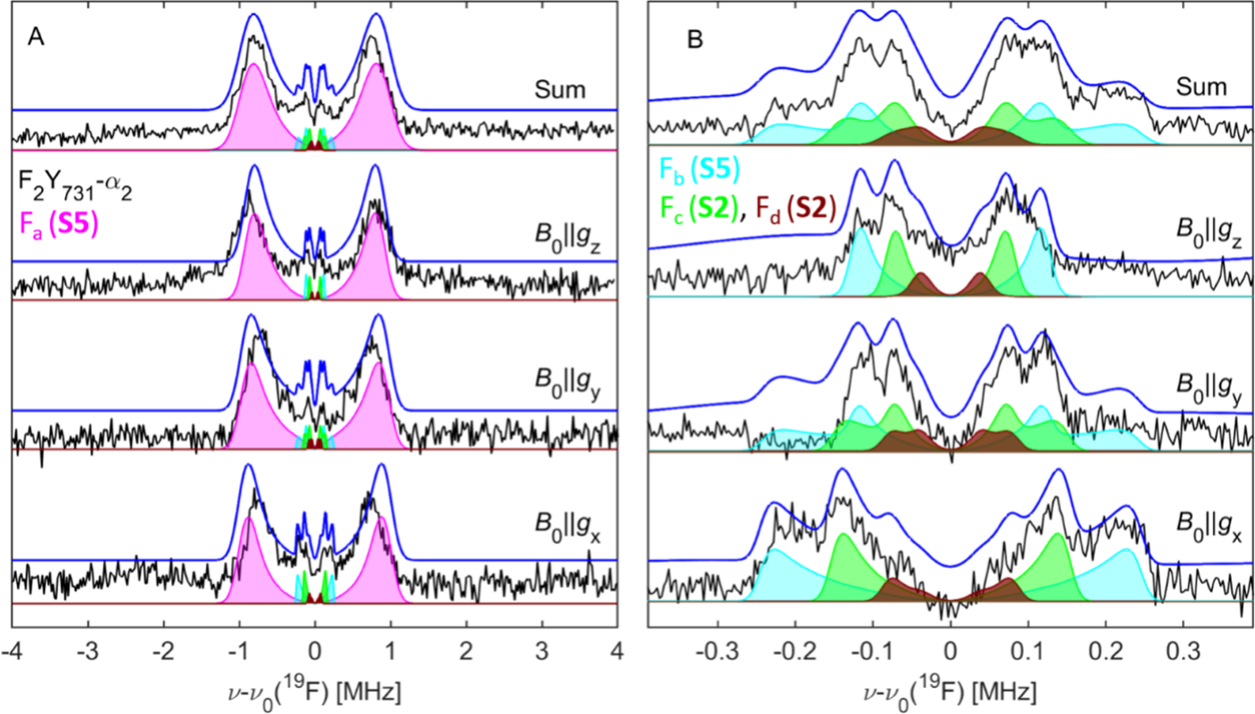
94 GHz ^19^F Mims ENDOR spectra on F_3_Y_122_-β_2_/F_2_Y_731_-α_2_ (80 μM, *T*_Q_ = 50 s, black lines) at *T* = 50 K. (A) Measurement with short τ values (∼250 ns). (B) Measurement with larger τ values (∼620 ns). Simulations including four different ^19^F atoms (F_a_–F_d_) are shown as blue lines and are based on **S2** and **S5** (Tables 1 and 2). Contributions of individual ^19^F atoms are shown as shaded areas: purple (F_a_), cyan, (F_b_), green (F_c_), and brown (F_d_).

Representative orientation-selected spectra, corresponding to the black sum spectra of Figure 5, are displayed in Figure 9. In the small coupling region (Figure 9B), we observe that *T*_∥_(F_b_) appears enhanced at *g*_x_, suggesting an orientation of the F_b_ dipolar tensor parallel to *g*_x_. Therefore, a structure similar to **S5** likely describes the data better than **S4**, as illustrated in Figure 7C,D, where the orientation of the dipolar vector with respect to *g*_x_ is displayed.

Using these orientational constraints, global simulations of the orientation-selective ENDOR spectra based on models **S2** and **S5** were carried out with the DFT-predicted parameters listed in Table 1 and the ratio (i.e., the relative contribution of **S2** and **S5**) varied until a minimum of residual could be found (SI 9). rmsd from these simulations for all samples amount to ca. 0.1 or 10% at the optimized ratios (Figure S18). We observed that the simulation of the large coupling F_a_ (Figure 9A) is not very sensitive to the weighting of **S2** and **S5**. This is expected as, under those experimental conditions, the resonances of F_b_–F_d_ are suppressed by the Mims blind spot in the center of the spectrum. Instead, the ratio F_b_/F_c_ affects the simulations of the small coupling region, as can be seen in Figure 9B by the decomposition of the simulation into the individual contributions. We note that the obtained weighting of the flipped conformation slightly varies between samples from 18 to 33% within an error of 5% for each sample (Table 2). Therefore, we estimate that the flipped conformation represents on average 25 ± 10% of the molecular ensemble.

**Table 1:** Parameters Used for the ENDOR Simulations

atom (model)	F–Y_356_^•^^a^ [Å]	*A*_x_, *A*_y_, *A*_z_^b^ [kHz]	*a*_iso_ [kHz]
F_a_ (**S5**)	4.6	580, −1668, −1952	–1013
F_b_ (**S5**)	7.3	–246, −246, 492	0
F_c_ (**S2**)	8.4	–159, −159, 318	0
F_d_ (**S2**)	10.0	–83, −83, 166	0

a Distances defined with respect to the centroid of Y_356_^•^, as shown in Figures 6 and 7B.

b Coupling constants *A*_*i*_ consider the anisotropic and the isotropic coupling constants (*T*_*i*_ and *a*_iso_, respectively): *A*_*i*_ = *T*_*i*_ + *a*_iso_. Euler angles for relating the *A* to *g* tensors are reported in Table S8. An error of ±15 kHz was estimated for couplings <500 kHz, while an error of ±125 kHz is estimated for the 1.6 MHz coupling (ca. 50% of the ENDOR line width parameter in both cases).

**Table 2:** Ratios of the Stacked Model **S2** and the Flipped Model **S5** from ENDOR Simulations

RNR mutant	*T*_Q_ [s]	contribution of flipped (**S5**)^a^
F_3_Y_122_-β_2_/F_2_Y_731_-α_2_	50	33%
F_3_Y_122_-β_2_/F_2_Y_731_-α_2_	143	22%
E_52_Q/F_3_Y_122_-β_2_/F_2_Y_731_-α_2_	35	18%
E_52_Q/F_3_Y_122_-β_2_/F_2_Y_731_-α_2_	153	25%

a Estimated error: ±5%; see Figure S18.

The representative best simulation for one sample F_3_Y_122_-β_2_/F_2_Y_731_-α_2_ is superimposed on the experimental data in Figure 9. Remarkably, the simulation of the orientation-selective spectrum at *B*_0_ ∥ *g*_*x*_ captures the selectivity for *T*_∥_(F_b_) and *T*_∥_(F_c_) and also reproduces the shoulders on the inner side, which were tentatively assigned to F_d_ in the discussion of Figure 5C. Given the challenges of the simulation procedure, we find that the obtained simulation reproduces the experimental data very satisfactorily.

### 3.3 ^17^O ENDOR with (E_52_Q)-F_3_Y_122_-β_2_/^17^O–Y-*wt*-α_2_

An independent effort was made to obtain experimental evidence for a flipped Y_731_ conformation in the trapped complex. We investigated whether a ^17^O ENDOR signal might be observable with a sample prepared using uniformly labeled ^17^O–Y-*wt*-α_2_ (^17^O in the phenol groups). This experiment was motivated by our recent successful observation of a ^17^O ENDOR signal from water H-bonded to Y_356_^•^.^28^ DFT calculations predicted a ^17^O–Y_731_–Y_356_^•^ coupling of ∼0.5 MHz for the flipped structure **S5**, slightly smaller than observed for H-bonded ^17^OH_2_ (0.7 MHz) (Table S9). We further considered issues that might make detection of this interaction more challenging. ^17^O has a lower gyromagnetic ratio than ^19^F (γ(^19^F)/γ(^17^O) ≈ 6.95) and its quadrupolar coupling may lead to signal broadening. In addition, the ^17^O–Y_731_-α_2_ is only 35–40%-labeled based on the available ^17^O–Y used during expression (SI 6). A reference ENDOR signal, with a comparable concentration of predicted ^17^O spins in close proximity to Y_356_^•^ (i.e., ca 10–20 μM), is shown in Figure S15. Despite potential unexpected issues, we proceeded with the experiment as ^17^O should be a sensitive nucleus at short distances (≲3 Å) and the ^17^O–Y_731_ coupling for the stacked conformation should not be detectable, allowing us to test the flipped Y_731_ model. As shown in SI 6.2, we were not able to observe any ^17^O couplings in three independently prepared samples. We have considered several possible explanations for these observations that may be related either to the experiment or to the use of F*_n_*Y probes: (1) the ^17^O coupling might be smaller than the DFT prediction and not detectable; (2) F_2_Y_731_ could experience a different flipping ratio or rate of flipping relative to Y_731_; (3) the F_3_Y_122_^•^ used to initiate radical transfer in the experiment is likely reduced to its phenolate, not phenol as with Y_122_^•^, and could play a role for the subunit interaction. These scenarios will be further discussed in the next section.

## 4 Discussion

In this paper, we report the use of 94 GHz ^19^F ENDOR spectroscopy, which has provided new insight into the chemistry of RT between Y_356_(β) and Y_731_(α) of *E. coli* RNR located at the subunit interface (Figure 2C,D). Success was possible using enzymes with site-specifically incorporated F*_n_*Ys: F_3_Y_122_-β_2_ (or E_52_Q/F_3_Y_122_-β_2_) and F_2_Y_731_-α_2_, which, when incubated with substrate (GDP) and effector (TTP), allowed trapping of the Y_356_^•^ pathway radical in an “active” α_2_β_2_ complex during the reverse RT pathway process. PELDOR and HF-EPR analysis established the location of the trapped radical, and the double mutant provided a direct link to the recent cryo-EM structure.^38^ The studies allowed measurement of the ^19^F–Y_731_ hyperfine couplings to Y_356_^•^, which report on their interspin distances and provide interesting mechanistic implications.

Analysis of 94 GHz ^19^F ENDOR spectra of the Y_356_^•^ required careful evaluation and subtraction of ^19^F signals associated with unreduced F_3_Y_122_^•^ and ^1^H backgrounds. Nevertheless, comparison of the spectra acquired at 50 and 80 K allowed unambiguous assignment of three distinct couplings between F_2_Y_731_ and Y_356_^•^.

Construction of small models of the three Ys and their DFT-predicted ^19^F HFC couplings, ENDOR orientation selection, and spectral simulations indicated that the ^19^F spectra are consistent with a mixture of flipped and stacked conformations of F_2_Y_731_ with respect to Y_730_, with flipped contributions of 25 ± 10% among the samples. While the flexibility of Y_731_ has been reported previously, the present results provide the first evidence for a conformation, in which the two pathway residues are located at an O–O distance of ∼3 Å, with potentially important consequences for understanding the interfacial PCET step. The presence of both conformations simultaneously suggests that they are energetically similar and may exist in equilibrium.

A number of different types of experiments have previously reported multiple Y_731_ conformations.^26,30^ In one study, in which CDP/ATP was incubated with *wt*-β_2_/R_411_A-NH_2_Y_731_-α_2_, an NH_2_Y_731_^•^ intermediate trapped in the forward RT was observed.^26^ The flipping was detected by PELDOR spectroscopy by its unusual Y_356_^•^/NH_2_Y_731_^•^ distance. This distance, however, was only observed in conjunction with an additional mutation at α-R_411_A. This residue sits in the α/β interface. In addition, transient absorption experiments in solution using the same α-R_411_A mutation and a photo-oxidant indicated a *k*_PCET_ between Y_356_F-photoβ_2_ and Y_731_ much faster than dNDP formation, ∼10^4^ s^–1^ versus 1–10 s^–1^.^30^

On the other hand, neither in the cryo-EM structure with E_52_Q/F_3_Y_122_-β_2_ nor in the ^17^O ENDOR experiments, which both employed F_3_Y_122_^•^ and *wt*-α_2_, was the flipped conformation of Y_731_ observed. Thus, while the role of F_2_Y_731_ in potentiating flipping is still unclear, the F_3_-phenolate generated at residue 122 during RT may not be the basis for a flipped Y_731_ conformation. In addition, the conditions for freeze-quenching the cryo-EM and ENDOR samples are very distinct in terms of protein concentration and glycerol content. A protein concentration of ∼80 μM had to be used for EPR samples, exceeding physiological RNR concentrations (ca. 1 μM). At elevated protein concentrations, the formation of α_4_β_4_ complexes has been reported.^69,70^ However, these complexes are incapable of producing Y_356_^•^ and should not affect the analysis of EPR experiments, in which Y_356_^•^ was observed selectively.

Overall, the complex interplay between Y_356_(β), Y_731_(α), R_411_(α), and other residues at the subunit interface is likely to be crucial for regulating the communication between the two redox-active Ys across the α/β interface.

Inspecting the predicted HFC parameters of the phenolic proton of F_2_Y_731_ with respect to Y_356_^•^ is another interesting source of information. The DFT calculations predicted HFCs of ∼6 MHz in models **S4** and **S5**. It is important to rationalize this finding in the context of previous ^1^H ENDOR studies on H-bond interactions to Y_356_^•^.^27^ In those studies, a ^1^H coupling in the range of 6 MHz was observed and assigned to one (or 2 equiv) H-bonded water molecule(s). The presence of the second water molecule was postulated to explain the unprecedented low *g*_x_ value of Y_356_^•^, i.e., 2.0062.^27^ The sharp peaks observed in our recent 263 GHz ^17^O ENDOR experiments support the presence of only a single water molecule.^28^ Given the similarity of coupling constants for the H-bonded protons for Y_731_ from either model **S4** or **S5**, the flipped conformation provides an explanation for the ^1^H coupling consistent with these previous ^1^H ENDOR data. To date, however, no ENDOR study has provided information on the interplay between stacked/flipped Y_731_ and the water binding at Y_356_^•^, which may be a key feature to control PCET across the interface. Interestingly, no distribution of *g*_*x*_ values at Y_356_^•^ is observed, indicating that the electrostatic environment is well defined and similar in both Y_731_ conformations. A mechanism, by which Y_731_ replaces a water molecule as a H-bond donor to Y_356_^•^ upon flipping, could explain this finding.

### 4.1 Implication of Flipped Y_731_ in PCET across α/β

Observation of flipped F_2_Y_731_ in close distance to Y_356_^•^, trapped in an active RNR complex, enables the examination of a mechanism for the PCET step between Y_356_^•^ and Y_731_ for the first time.

The current hypothesis for interfacial PCET involving water, as noted above, was based on the ENDOR studies and the H-bond to Y_356_^•^ assigned to water.^27,28^ Recent MD simulations^45^ based on the cryo-EM structure supported the role of water first suggested by Nick et al.^27^ The simulations additionally showed that water molecules can be present at the α/β interface including between Y_356_ and Y_731_, between Y_356_ and β-E_52_ (an interface residue), and support a pathway for water to escape to the bulk solvent.^38,45^ Interestingly, MD also revealed an equilibrium between flipped and stacked conformation for Y_731_, both populated at room temperature.^45^ Nevertheless, the reported flipped Y_731_ structure from the MD study still shows a long O–O distance to Y_356_ (∼8 Å on average), precluding a direct interaction between the two Ys.^45^

Thus, the mechanism of PCET between Y_356_ and Y_731_ (i.e., during reverse and forward RTs) remained to be resolved due to the long Y_356_–Y_731_ distance (∼8 Å) observed in the cryo-EM structure.^38^ We note that the published cryo-EM structure and ENDOR data have distinct problems. The resolution of the cryo-EM structure was insufficient to resolve waters. The ENDOR studies only detected water in the first coordination sphere of Y_356_^•^, i.e., in a distance range of ∼3 Å.^27,28^

The ^19^F ENDOR data presented here, despite the issues raised, provide evidence for close interaction between the two Ys across the subunit interface in an active RNR construct. In our ENDOR-derived model **S5**, the O–O distance between Y_356_^•^–Y_731_ amounts to 3.0 ± 0.2 Å, with a similar value in the related model **S4**. This distance is within the range of the distances reported for the pathway pair C_439_–Y_730_ (O–S: 3.7 Å in the X-ray structure of α_2_ versus 3.4 Å in α-NH_2_Y_730_)^13,44^ as well as for the pair Y_730_–Y_731_ (O–O: 3.3 Å in α_2_ versus 2.7 Å in α-NH_2_Y_730_).^13,44^ For these pairs, independent quantum chemical calculations predicted a colinear PCET mechanism,^24,71,72^ in which the electron and proton are transferred individually in one step from the same donor to the same acceptor, although a water-assisted PCET has been proposed and discussed for the C_439_–Y_730_ pair.^73^ Recently, also an alternative, glutamate (E_623_)-mediated proton transfer for the RT between Y_731_ and Y_730_, has been proposed based on MD simulations and QM/MM analysis.^74^ A key conclusion from the latter study based on the analysis of E_623_ was that forward and reverse RTs are different. Interestingly, our earlier large-scale DFT calculation on the pathway triad C_439_–Y_730_–Y_731_ predicted that the coordination of a water molecule to Y_730_^•^ can stabilize this radical intermediate and the transition states to the next pathway intermediates, Y_731_^•^ and C_439_^•^.^24^ Therefore, the calculation pointed to a functional role of water in PCET without its direct involvement as a proton donor or acceptor. Based on these considerations, we propose that our current results are consistent with a model of colinear PCET mechanism for the RT Y_356_^•^(β) – Y_731_(α) ⇄ Y_356_(β) – Y_731_^•^(α). This mechanism requires a conformational change of Y_731_ during the long-range RT, as the next step (Y_731_^•^(α) – Y_730_(α) ⇄ Y_731_(α) – Y_730_^•^(α)) occurs in the stacked conformation of the Y_731_/Y_730_ pair.

## 5 Conclusions

Use of site-specifically incorporated unnatural amino acids and kinetic trapping in conjunction with high-field ENDOR, PELDOR, and EPR spectroscopies has given new insight into the PCET involving Y_356_^•^(β) and Y_731_(α) across the RNR subunit interface. ^19^F ENDOR revealed two sets of hyperfine coupling constants for F_2_Y_731_ caused by the occurrence of two distinct conformations. One set of hyperfine couplings is consistent with a stacked Y_731_ conformation at an ∼8 Å distance (O–O) to Y_356_^•^, as observed by cryo-EM. However, much larger ^19^F couplings revealed a second conformation, in which F_2_Y_731_ is flipped toward Y_356_^•^ at a much shorter O–O distance of ∼3 Å. This distance is similar to distances between other Y pairs on the RT pathway in α, for which colinear PCET has been established.

These results reveal again the ability and importance of EPR spectroscopic methods and new experimental designs for the detection of multiple conformations in a biological machinery.

## Supporting Information Available

Measurement of RNR activity, EPR sample preparation, EPR experiments (263, 34, and 94 GHz spectroscopies including relaxation measurements, radical yield determination, and background corrections), ^17^O ENDOR data, modeling of the tyrosine triad, and ENDOR simulations (PDF)

## Notes

Original spectroscopic data associated with Figures 5 and 9 as well as all xyz coordinates of the model structures can be accessed via the open database Göttingen Research Online (https://doi.org/10.25625/YXHC63).
