# Supplementary material for: 19F Electron-Nuclear Double Resonance Reveals Interaction between Redox-Active Tyrosines across the α/β Interface of E. coli Ribonucleotide Reductase
Source: J Am Chem Soc. 2022 Jun 2;144(25):11270–82. doi: 10.1021/jacs.2c02906 (PMC9248007; doi:10.1021/jacs.2c02906)
Supplement: Supplementary file 1 — ja2c02906_si_001.pdf [file ja2c02906_si_001.pdf]

^{19}F Electron Nuclear Double Resonance reveals interaction between redox active tyrosines across the α/β interface of *E. coli* ribonucleotide reductase

Andreas Meyer, Annemarie Kehl, Chang Cui, Fehmke A. K. Reichardt, Fabian Hecker, Lisa-Marie Funk, Kuan-Ting Pan, Henning Urlaub, Kai Tittmann, JoAnne Stubbe, Marina Bennati

Content

1. Measurements of RNR activity 1
2. EPR sample preparation 3
3. 263 GHz EPR spectroscopy 5
4. PELDOR experiments 7
4.1. 34 GHz (Q-band) EPR spectra 7
4.2. 34 GHz (Q-band) measurements of the phase memory time T_M 7
4.3. 34 GHz (Q-band) PELDOR 8
5. 94 GHz EPR and ENDOR measurements of ^{19}F labelled RNR 11
5.1. EPR measurements at 50 K 11
5.2. Relaxation measurements at 50 K 11
5.3. Choosing τ values for ^{19}F ENDOR measurements 12
5.4. Background correction in 50 K ^{19}F ENDOR spectra 14
5.5. EPR measurements at 80 K 16
5.6. ^{19}F ENDOR at 80 K and comparison to 50 K 17
6. Control experiments with ^{17}O-Y-α_2 18
6.1. Preparation of ^{17}O-Y-α_2 and mass spectrometric determination of labelling degree 18
6.2. ^{17}O ENDOR 20
7. DFT Calculations for the simulations of the ENDOR spectra 21
8. Models of Y_{356}•, F_2Y_{731}, and Y_{730} 23
9. Optimizing the stacked/flipped ratio in simulations of ENDOR spectra for different samples 26
10. References 27
Appendix. XYZ coordinates of models S2 and S5 29

1. Measurements of RNR activity

The specific activity SA of RNR proteins was measured by the spectrophotometric assay[1,2] using a Cary 100 UV-Vis spectrometer (Agilent) at 25 °C. The temperature was controlled by a circulating water flow bath.

The influence of glycerol on wt-RNR activity was measured as following. The final concentrations in the assay amounted to 0.75 µM for *wt*-α_2, 0.15 µM for *wt*-β_2, 80 µM *E. Coli* thioredoxin (TR), 0.5 µM, *E. Coli* thioredoxin reductase (TRR), 1 mM CDP, and 3 mM ATP in assay buffer (50 mM HEPES, 15 mM $MgSO_4$, 1 mM EDTA, pH 7.6) and glycerol contents of 0, 9, 18 and 27 v%. Practically, the concentrations were established in the cuvette by mixing 200 µL of a mastermix containing all ingredients except *wt*-β_2 at twice the concentration in assay buffer with an equal volume of assay buffer premixed with adequate amounts of glycerol. The reaction was initiated by first adding a volume of NADPH solution (ca. 7 µL, 11.8 mM) and then, after measuring background NADPH consumption, 4 µL of 15 µM *wt*-β_2-RNR (1.2 Y_{122}•/β_2).[1] For each glycerol content, at least three runs were performed. The results are summarized in Figure S1 and Table S1. The SA values agree with reported *wt*-activities[1,2] and indicate that the high glycerol contents used for EPR sample preparation do not preclude activity of RNR.

The procedure for measuring the SA of $E_{52}QF_3Y_{122}$-β_2/F_2Y_{731}-α_2 and F_3Y_{122}-β_2/F_2Y_{731}-α_2 was similar to the procedure described above, the results are summarized in Table S1. $E_{52}QF_3Y_{122}$-β_2/F_2Y_{731}-α_2 solution shows no SA within error. F_3Y_{122}-β_2/F_2Y_{731}-α_2 on the other hand clearly shows SA amounting to 6.8 ± 0.1% of the *wt*-enzyme. This value is in the expected range, given that the activities of *wt*-β_2/F_2Y_{731}-α_2 and F_3Y_{122}-β_2/*wt*-α_2 have been reported as 60 ± 10%[3] and 20 ± 10%,[1,4,5] respectively.

Table S1. Activities of investigated RNR mutants.

RNR mutant	V% glycerol	Specific activity [nmol/mg·min]
wt	0	8200 ± 370
wt	9	6400 ± 140
wt	18	4300 ± 80
wt	27	2700 ± 100
F_3Y_{122}-β_2/F_2Y_{731}-α_2	0	560 ± 10
β_2/F_2Y_{731}-α_2[3]	5	1400 ± 100[a, b]
F_3Y_{122}- β_2/α_2[4]	5	1300 ± 100[b]
$E_{52}QF_3Y_{122}$-β_2/F_2Y_{731}-α_2	0	0 ± 10
$E_{52}QF_3Y_{122}$-β_2/ α_2[1]	5	6[b,c]

[a] The SA of the α_2 subunit was assayed. [b] Assay conditions are detailed in the corresponding references.
[c] The reported residual SA was potentially caused by traces of co-expressed *wt*-β_2.

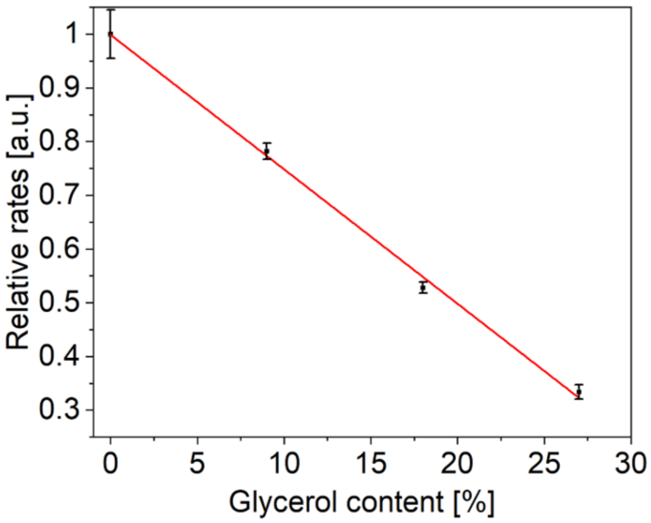

Figure S1. Relative NADPH consumption rates of *wt*-RNR turnover as a function of glycerol content, normalized to the highest observed rate (Table S1).

2. EPR sample preparation

The most important samples have been described in the main text. Here, we give information on additional samples. All samples are summarized in Table S2.

In addition to the W-band samples containing glycerol as described in the main text, also samples with faster freezing points (T_Q =11 – 13 s) have been prepared without glycerol. These samples could be used to record EPR spectra at W-band, but the phase memory time T_M was found to be too short for sensitive ENDOR measurements. We also tried to prepare samples in which the α subunit (along with effector and substrate) was premixed with glycerol to achieve considerably faster quenching. However, this procedure led to visible precipitation of protein after mixing with the β subunit, even though premixing with glycerol was possible under the conditions of the spectrophotometric assay.

For Q-band PELDOR measurements, samples with RNR subunit concentrations of only 40 µM have been prepared in an analogous fashion as the samples at ~100% higher concentration described in the main text. Furthermore, $E_{52}QF_3Y_{122}$-β_2/*wt*-α_2 (150 µM, 20v% glycerol) was prepared for an additional reference measurement.

For measuring ^{1}H background contributions of Y_{356}•, ENDOR spectra of F_3Y_{122}-β_2/Y_{730}F-α_2 (95 µM, 20v% glycerol) were recorded. The sample was prepared in a 0.5 mm inner diameter (ID) suprasil tube analogously to the other samples.

For measuring ^{19}F background contributions of F_3Y_{122}• to the ENDOR spectra, F_3Y_{122}-β_2 (300 µM, 33v% glycerol) was prepared in a 0.5 mm ID suprasil tube.

For performing W-band ^{17}O ENDOR control experiments, Y_{356}• was trapped using either F_3Y_{122}-β_2 or $E_{52}QF_3Y_{122}$-β_2 in combination with ^{17}O-Y-α_2 in an analogous fashion to the other samples in 0.5 mm ID suprasil tubes (concentrations and T_Q values are listed in Table S2).

Table S2. List of EPR samples prepared in this work. Estimated errors amount to ±5 % for the concentrations and ±3 s for T_Q.

RNR Construct	Subunit Conc. [μM]	MW band[a]	Quenching time T_Q [s]	Glycerol content [%]	Experiments
$E_{52}QF_3Y_{122}$-β_2/F_2Y_{731}-α_2	80	W	35	20	EPR, ENDOR
$E_{52}QF_3Y_{122}$-β_2/F_2Y_{731}-α_2	80	Q	44	20	EPR, PELDOR
$E_{52}QF_3Y_{122}$-β_2/F_2Y_{731}-α_2	40	Q	49	20	EPR, PELDOR
$E_{52}QF_3Y_{122}$-β_2/F_2Y_{731}-α_2	80	W	153	20	EPR, ENDOR
F_3Y_{122}-β_2/F_2Y_{731}-α_2	80	W	50	20	EPR, ENDOR
F_3Y_{122}-β_2/F_2Y_{731}-α_2	80	Q	77	20	EPR, PELDOR
F_3Y_{122}-β_2/F_2Y_{731}-α_2	40	Q	69	20	EPR, PELDOR
F_3Y_{122}-β_2/F_2Y_{731}-α_2	80	W	143	20	EPR, ENDOR
F_3Y_{122}-β_2/F_2Y_{731}-α_2	80	Q	166	20	EPR, PELDOR
F_3Y_{122}-β_2	300	W	-	33	EPR, ENDOR
F_3Y_{122}-β_2/$Y_{730}F$-α_2	95	W	51	20	EPR, ENDOR
$E_{52}QF_3Y_{122}$-β_2/*wt*-α_2	150	Q	50	20	EPR, PELDOR
F_3Y_{122}-β_2	200	263	-	0	EPR
F_3Y_{122}-β_2/F_2Y_{731}-α_2	170	263	20	0	EPR
$E_{52}QF_3Y_{122}$-β_2/F_2Y_{731}-α_2	170	263	120	0	EPR
F_3Y_{122}-β_2/wt-α_2	190	263	14	0	EPR
F_3Y_{122}-β_2/^{17}O-Y-α_2	80	W	32	20	EPR, ENDOR
F_3Y_{122}-β_2/^{17}O-Y-α_2	110	W	37	20	EPR, ENDOR
$E_{52}QF_3Y_{122}$-β_2/^{17}O-Y-α_2	135	W	37	20	EPR, ENDOR

[a] MW band designates the approximate microwave frequency and field strength. Q corresponds to ~34 GHz/1.2 T, W to ~94 GHz/3.4 T, and 263 to ~263 GHz/9.4 T.

3. 263 GHz EPR spectroscopy

263 GHz EPR measurements were performed on a Bruker ELEXSYS E780 spectrometer equipped with a 100 mW Amplifier-Multiplier-Chain (AMC, Virginia Diodes Inc.) coupled to the EPR/ENDOR resonator E950100 (Bruker BioSpin) via a corrugated waveguide, achieving $\pi/2$ pulse lengths of 30 – 34 ns. Two-pulse echo detected EPR experiments were performed at 80 K, where contributions from unreacted F_3Y_{122}• are strongly suppressed.[6] τ values were 300 ns. Shot repetition times amounted to 3 ms. The derivatives of the echo detected EPR spectra was calculated after data smoothing using 4th order Savitzky-Golay filtering (35 – 55 points). Additional information on the high-frequency set-up is described in a recent publication.[12]

Echo detected EPR spectra at 263 GHz are presented in Figure S2 A and B, where each spectrum contains contributions from $F_3Y_{122}^•$ and $Y_{356}^•$. The contribution of $Y_{356}^•$ to the spectra in Figure S2 varies due to different radical yields in the different samples. As a representative example, Figure S3 illustrates the decomposition of the spectrum of F_3Y_{122}-β_2/F_2Y_{731}-α_2 into its contributions, which is achieved by subtraction of a reference spectrum of $F_3Y_{122}^•$.

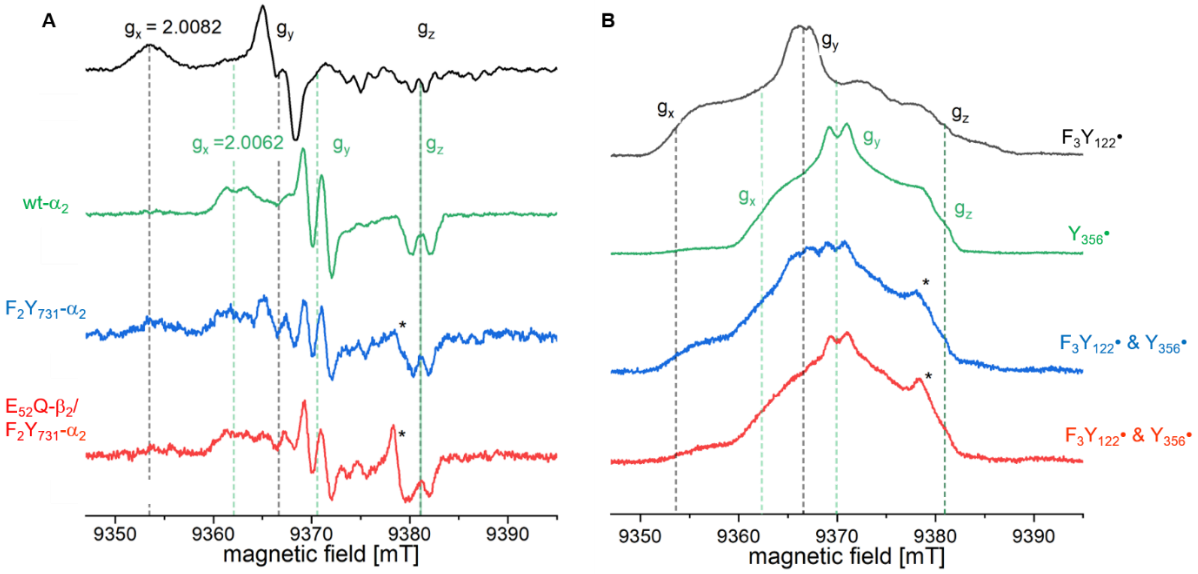

Figure S2. Echo-detected EPR spectra (**A**: derivative spectrum, **B**: original, absorption signal) obtained at 263 GHz and T = 80 K of F_3Y_{122}-β_2 (black line, shot-repetition-time = 3 ms, shots-per-point = 512, 65 scans), F_3Y_{122}-β_2/wt-α_2 (green line), F_3Y_{122}-β_2/F_2Y_{731}-α_2 (blue line, shot-repetition-time = 3 ms, shots-per-point = 256, 47 scans), and $E_{52}QF_3Y_{122}$-β_2/F_2Y_{731}-α_2 (red line, shot-repetition-time = 3 ms, shots-per-point = 256, 55 scans). The asterisk marks a background signal due to paramagnetic defect centers in the quartz glass of the EPR tubes.[13]

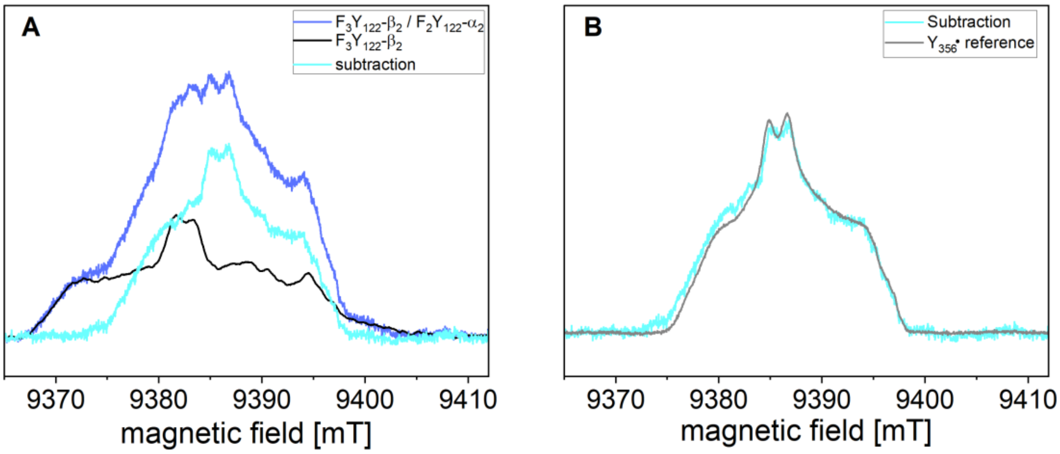

Figure S3. Decomposition of the spectra of F_3Y_{122}-β_2/F_2Y_{731}-α_2 (blue line, 80 K) into its contributions. A) Subtraction of the spectrum of F_3Y_{122}• measured at 80 K, shown in black. The black spectrum was measured with the same measurement parameters as the blue one. B) Comparison of the subtraction with Y_{356}• reference spectrum in grey (reference obtained on F_3Y_{122}-β_2/wt-α_2 at 80 K).

4. PELDOR experiments

4.1. 34 GHz (Q-band) EPR spectra

Q-band EPR spectra at 10 K allow quantifying the yield of trapped Y_{356}• as demonstrated on one representative example in Figure S4. Y_{356}• yields in the F_3Y_{122}-β_2/F_2Y_{731}-α_2 samples turned out lower (typically 15 – 20 %) than in the $E_{52}Q$ samples (typically 30 – 35 %). Both are within the typical range observed previously (20 – 40 %).[6–8] It is noted, that the samples for Q- and W-band measurements were prepared as one batch, leading to basically identical radical yields for Q- and W-band samples.

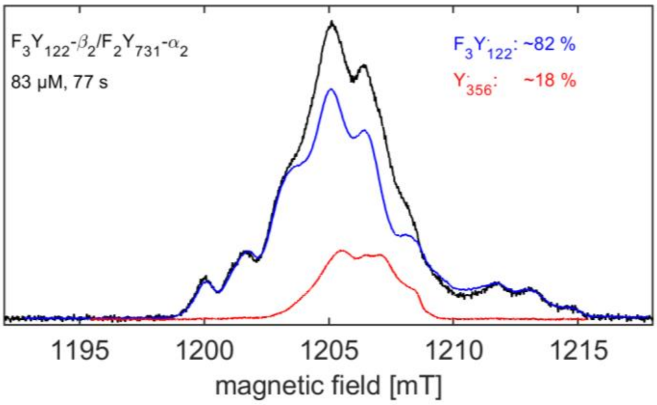

Figure S4. Representative example of a Q-band EPR spectrum of an F_3Y_{122}-β_2/F_2Y_{731}-α_2 sample recorded at 10 K (black lines) along with a comparison to F_3Y_{122}• and Y_{356}• reference spectra (blue and red lines, respectively). The reference spectra were obtained as described earlier.[6]

4.2. 34 GHz (Q-band) measurements of the phase memory time T_M

T_M measurements of Y_{356}• at Q-band and T = 50 K were performed by measuring the echo intensity as a function of the interpulse separation τ at three observer positions O1, O2, and O3 marked in Figure S5, which were also used for subsequent PELDOR experiments. The echo intensity I as function of τ could be fit satisfactorily using a biexponential decay function (Equation S1):

$$I = A_1 e^{-2\tau/T_{M,1}} + A_2 e^{-2\tau/T_{M,2}} \quad \text{(S1)}$$

The biexponential fitting function allows for contributions of two radical species, as F_3Y_{122}• is detectable in all samples at this temperature. The obtained fitting parameters are tabulated in Table S3. Notably, $T_{M,2}$ was slightly higher for the F_3Y_{122}-β_2/F_2Y_{731}-α_2 samples, which allowed for longer observation windows (see Figure 4, main text).

Table S3. Typical fitting parameters (eq. S1) of the echo decay curves.

Sample	Obs. Pos.	A_1[a]	$T_{M,1}$[a] [µs]	A_2	$T_{M,2}$ [µs]
$E_{52}QF_3Y_{122}$-β_2/F_2Y_{731}-α_2	**1**	**-**	**-**	**1**	**2.25**
$E_{52}QF_3Y_{122}$-β_2/F_2Y_{731}-α_2	**2**	**-**	**-**	**1**	**2.12**
$E_{52}QF_3Y_{122}$-β_2/F_2Y_{731}-α_2	**3**	**-**	**-**	**1**	**2.07**
F_3Y_{122}-β_2/F_2Y_{731}-α_2	**1**	**-**	**-**	**1**	**2.38**
F_3Y_{122}-β_2/F_2Y_{731}-α_2	**2**	**0.21**	**0.14**	**0.79**	**2.21**
F_3Y_{122}-β_2/F_2Y_{731}-α_2	**3**	**0.27**	**0.19**	**0.73**	**2.23**

[a] In some cases, the fitting resulted in a monoexponential decay curve.

4.3. 34 GHz (Q-band) PELDOR

Since orientation selectivity is observed in PELDOR measurements on tyrosyl radicals in RNR, three time traces with different observer and pump positions (Figure S5) were recorded and summed up to reduce the effects of orientation selectivity on the analysis of the time traces. An example for the resulting orientation selective PELDOR time traces is shown in Figure S5.

Figure S5 shows the sum time traces of seven RNR samples with trapped Y_{356}• along with their analysis using Tikhonov regularization as implemented in DeerAnalysis 2019.[9,10] Six of the examples are obtained using the F_2Y_{731}-α_2 subunit, which was also used for ^{19}F ENDOR. Regardless of concentration, T_Q, and identity of the β-subunit, all samples yield indistinguishable distance distributions. The larger width of the E_{52}Q double mutants is a result of the shorter observation window, and this difference disappears if the time traces of the F_3Y_{122}-β_2/F_2Y_{731}-α_2 samples truncated at the same time point. An additional reference time trace was obtained using $E_{52}QF_3Y_{122}$-β_2/*wt*-α_2 which resulted in the same single distance of ~3.0 nm in the distance distribution.

The results of all PELDOR measurements are summarized in Table S4.

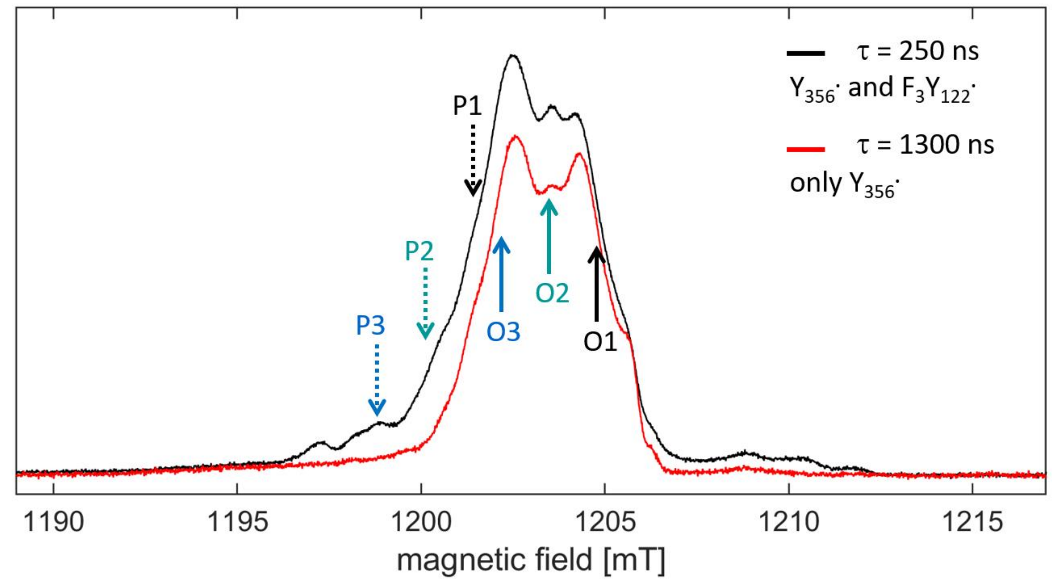

Figure S5. EPR spectrum of $E_{52}QF_3Y_{122}$-β_2/F_2Y_{731}-α_2 (80 µM, 135 s) with pump (dashed arrows, labels P1 – P3) and observer positions (full arrows, labels O1 – O3) for orientation selective PELDOR measurements. Both displayed EPR spectra are obtained from the same sample at a temperature of 50 K, but with different τ values. The red spectrum is scaled to approximately match the Y_{356}• contribution in the black spectrum. At 50 K, F_3Y_{122}• contributes markedly to the EPR spectrum if small τ values are used, but is largely suppressed at larger ones owed to faster relaxation of F_3Y_{122}•.

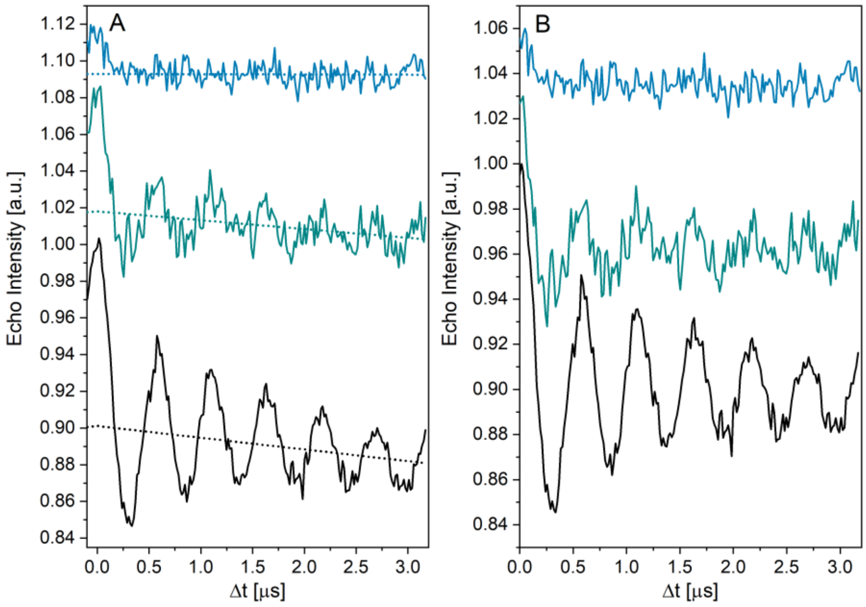

Figure S6. A) Example of orientation selective PELDOR time traces (sample F_3Y_{122}-β_2/F_2Y_{731}-α_2) at 50 K. The color code indicates the observer position of the time traces and corresponds to the color code used in Figure S5. Number of scans are 1300, 680 and 938 for the black, green and blue time traces, respectively, corresponding to measurement times of 15, 8 and 11 h. The background functions are shown as dotted lines. B) Time traces after background correction.

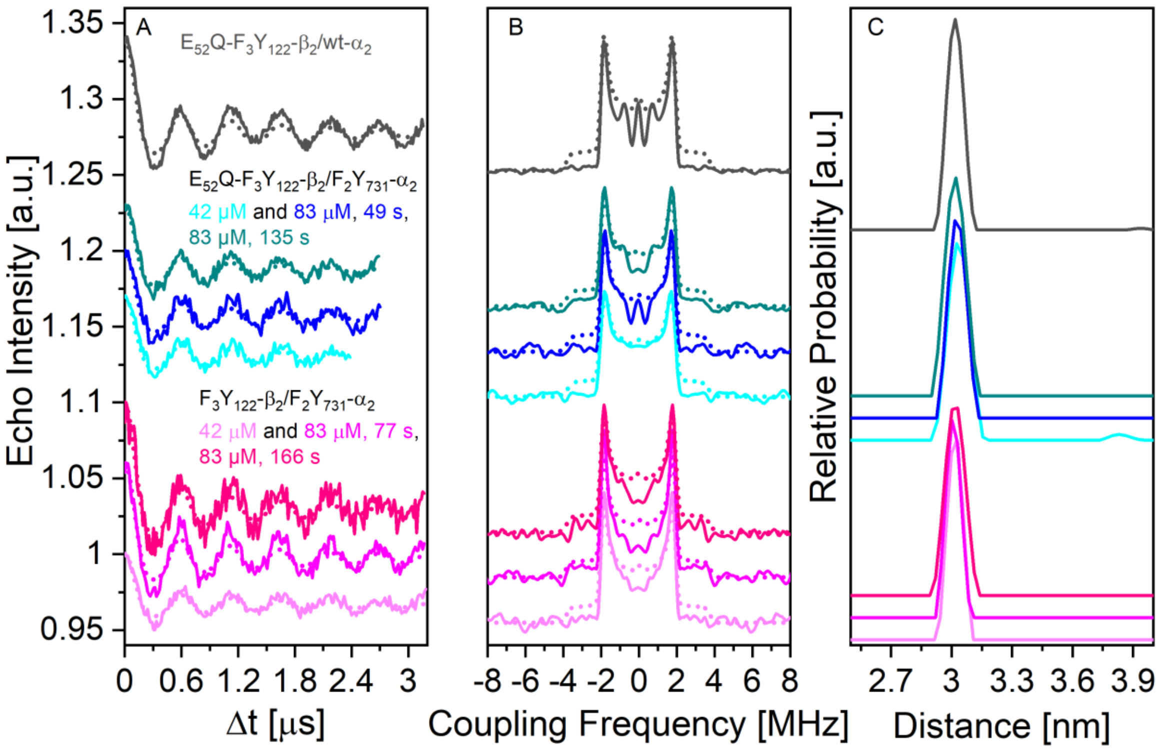

Figure S7. A) Orientation averaged and background corrected PELDOR time traces of seven different samples measured at 50 K. B) Fourier transforms of the traces from A). C) Distance distributions. The dashed lines in A) and B) show the simulation of the data obtained using DeerAnalysis.

Table S4. Summary of the analysis on the PELDOR traces using Tikhonov regularization and the L-curve criterion of the Q-band PELDOR time traces.

RNR Mutant	Subunit Conc. [μM]	Freezing time [s]	Main distance[a] [nm]	FWHM[b] [nm]
$E_{52}QF_3Y_{122}$-β_2/F_2Y_{731}-α_2	80	44	3.03 ± 0.02	0.13 ± 0.03
$E_{52}QF_3Y_{122}$-β_2/F_2Y_{731}-α_2	40	49	3.03 ± 0.02	0.13 ± 0.03
$E_{52}QF_3Y_{122}$-β_2/F_2Y_{731}-α_2	80	135	3.02 ± 0.02	0.14 ± 0.03
F_3Y_{122}-β_2/F_2Y_{731}-α_2	80	77	3.02 ± 0.02	0.09 ± 0.03
F_3Y_{122}-β_2/F_2Y_{731}-α_2	40	69	3.02 ± 0.02	0.09 ± 0.03
F_3Y_{122}-β_2/F_2Y_{731}-α_2	80	166	3.02 ± 0.02	0.13 ± 0.03
$E_{52}QF_3Y_{122}$-β_2/wt-α_2	150	50	3.02 ± 0.02	0.10 ± 0.03

[a] Measured at the maximum of the main peak, error estimated by varying fit parameters in DeerAnalysis.
[b] FWHM = full width at half maximum. The width in all time traces is determined by the observation window (the oscillations are not fully damped at the end of the window). The given error estimate was chosen to reflect that the widths of all distributions are considered to be indistinguishable throughout the samples.

5. 94 GHz EPR and ENDOR measurements of ^{19}F labelled RNR

5.1. EPR measurements at 50 K

A representative echo detected EPR spectrum of an F_3Y_{122}-β_2/F_2Y_{731}-α_2 at 50 K with a short τ value of 240 ns is displayed in Figure S8. Under these conditions as well as under the conditions used for measuring the ~1.6 MHz ^{19}F HFC, both F_3Y_{122}• and Y_{356}• contribute to the EPR spectrum, as is also demonstrated in Figure S8. Under the conditions used for measuring the < 250 kHz ^{19}F HFCs, the contribution of F_3Y_{122}• is suppressed because of its faster relaxation compared to Y_{356}•.

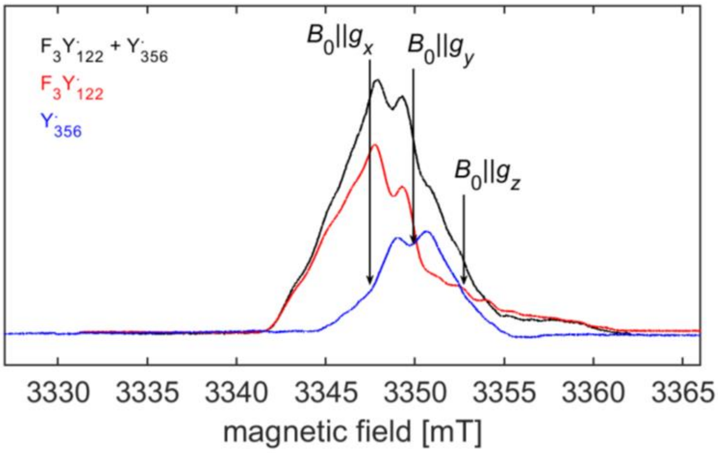

Figure S8. Representative example of a W-band EPR spectrum of an F_3Y_{122}-β_2/F_2Y_{731}-α_2 sample recorded at 50 K with τ = 240 ns (black lines) along with a comparison to spectrum of F_3Y_{122}• (red). The blue spectrum was obtained by subtraction (black minus red). A resonator background signal which amounted to < 10% of the total intensity was subtracted.

5.2. Relaxation measurements at 50 K

The spin lattice relaxation time T_1 of Y_{356}• was measured with the inversion recovery (IR) sequence. All samples used for ^{19}F ENDOR measurement yielded similar results. The IR curves were fitted using Eq. (S1), with two components in almost equal weights having time constants of 300 µs and 1650 µs. At 2000 µs, which is the value used as SRT throughout the ENDOR experiments for all samples, more than 85% of the signal is recovered.

For the phase memory time T_M, echo decay curves using the three-pulse echo sequence with a variety of T values were performed. T_M for each T value was estimated by fitting Equation (S1) to the resulting time traces. Table S5 summarizes representative results obtained on $E_{52}QF_3Y_{122}$-β_2/F_2Y_{731}-α_2 at 50 K.

Finally, we also tested some of the samples which were prepared without addition of glycerol. These samples had notably lower phase memory times (typically 25 – 30 % faster relaxation, data not shown), thereby yielding lower sensitivities despite having higher concentrations, which prohibited their use for ENDOR.

Table S5. Fitting parameters according to Eq. (S1) of the echo decay curves of $E_{52}QF_3Y_{122}$-β_2/F_2Y_{731}-α_2 (80 µM, T_Q = 153 s) at 50 K.

***T* [µs]**	**A_1**	**$T_{M,1}$ [ns]**	**A_2**	**$T_{M,2}$ [ns]**
1	**0.21**	**300**	**0.79**	**1930**
11	**0.09**	**171**	**0.91**	**1181**
21	**0.06**	**135**	**0.94**	**1000**
31	**-**	**-**	**1**	**866**
41	**-**	**-**	**1**	**816**
51	**-**	**-**	**1**	**782**
61	**-**	**-**	**1**	**752**
71	**-**	**-**	**1**	**725**
81	**-**	**-**	**1**	**739**
91	**-**	**-**	**1**	**698**
101	**-**	**-**	**1**	**699**

5.3. Choosing τ values for ^{19}F ENDOR measurements

The ^{19}F ENDOR spectra shown in this work consist of various contributions with vastly different coupling constants ranging from ~0.07 – 1.6 MHz. For the couplings ≲250 kHz, different parameters are needed than for the couplings of ~1.6 MHz. Here, our strategy for choosing τ values is described.

Couplings ≲250 kHz

The strategy for choosing τ when measuring small couplings aimed at high resolution while simultaneously avoiding the occurrence of a disturbing proton background signal.

τ-dependence of Mims ENDOR sensitivity. For the small couplings, the same procedure as in Ref. [11] was applied, and the sensitivity *S* of the ENDOR experiment was optimized using Equation (S2)[11]

$$S = F \cdot I \qquad \text{(S2)}$$

Equation (S2) takes into account the τ dependent echo intensity *I* and the ENDOR efficiency *F* (Equation S3):[11]

$$F = 0.5 \cdot \sin^2\left(2\pi \cdot \frac{A}{2}\tau\right) \qquad \text{(S3)}$$

T_M values on the order of 700 – 900 ns (Table S5) lead to a flat maximum in sensitivity around τ values of 600 – 750 ns for coupling constants of 150 – 250 kHz.

τ-Suppression of the ^{1}H couplings of Y_{356}•. Resonances from the protons at the 3- and 5-position of Y•, which have coupling constants on the order of 20 MHz,[12] are a second factor considered for the choice of τ. These protons lead to broad resonances around the ^{19}F Larmor frequency $\upsilon_{^{19}\mathrm{F}}$, which distort the ^{19}F spectra. It was assumed, that the least disturbing background for small couplings (≤ ~250 kHz) would result by choosing a τ value that leads to maximum ^{1}H ENDOR efficiency *F* (Eq. S3) at $\upsilon_{^{19}\mathrm{F}}$. Then, proton backgrounds that are symmetric around the ^{19}F Larmor frequency but could not be mistaken for ^{19}F resonances are expected, as these backgrounds have their maxima at the center of the ^{19}F spectrum and fall off on both sides of the ^{19}F Larmor frequency. Furthermore, ^{1}H resonances were expected to be broad and

featureless in the narrow region investigated for ^{19}F couplings of ≤ 250 kHz. τ values which lead to maximum ^{1}H ENDOR efficiency at $\upsilon_{^{19}\mathrm{F}}$ were calculated by reformulating Eq. (S3) and setting A = $\upsilon_{^{1}\mathrm{H}} - \upsilon_{^{19}\mathrm{F}}$, leading to Eq. (S4):

$$\tau = \frac{1}{2}\frac{1000(n+1/2)}{\upsilon_{^{1}\mathrm{H}}-\upsilon_{^{19}\mathrm{F}}} = \frac{500(n+1/2)}{\upsilon_{^{1}\mathrm{H}}-\upsilon_{^{19}\mathrm{F}}}\,\mathrm{MHz}\cdot\mathrm{ns} \qquad \text{(S4)}$$

In Equation (S4), *n* represents any integer number. The factor of 1000 considers the conversion factors of the frequency and time units (MHz and ns, respectively). Thus, choosing *n* = 10 in Eq. (S4) leads to τ ~620 ns. This value also allows for good sensitivity *S*.

Couplings of ~1.6 MHz

For the 1.6 MHz coupling, much shorter τ values had to be used and the proton background was more disturbing because of the larger RF window. Two ENDOR measurements with a τ value calculated either by Equation (S4) or by Equation (S5) were conducted.

$$\tau = \frac{500\cdot n}{\upsilon_{^{1}\mathrm{H}}-\upsilon_{^{19}\mathrm{F}}}\,\mathrm{MHz}\cdot\mathrm{ns} \qquad \text{(S5)}$$

τ values following Equation (S5) lead to minimum ENDOR efficiency *F* for the protons around $\upsilon_{^{19}\mathrm{F}}$. Choosing *n* = 4 in Equations (S4) and (S5) leads to τ values of ~ 266 and 236 ns, respectively. This small difference leaves the ^{19}F contributions to the ENDOR spectrum nearly unaffected but leads to a dramatic change of the ^{1}H background and allows identification of features belonging to the ^{1}H background. Furthermore, τ values of ~236 and 266 ns lead to a maximum sensitivity for ^{19}F couplings at ~ ±1 MHz, i.e. very close to the peaks caused by 1.6 MHz couplings. Blind spots are placed at ~ ±2 MHz with these τ values, which is far away from the observed resonances.

5.4. Background correction in 50 K ^{19}F ENDOR spectra

Two different background signals have to be considered in the ^{19}F ENDOR measurements shown in Figures 5 and 8. First, the ^{1}H background stemming from the 3,5-protons of Y_{356}•[6] and, second, ^{19}F resonances originating from unreacted 2,3,5-F_3Y_{122}•.[5] ^{1}H signals stemming from 2,3,5-F_3Y_{122}• are not expected near the ^{19}F Larmor frequency. Again, the measurements aiming at couplings $\lesssim$250 kHz or at couplings of ~1.6 MHz have to be treated differently.

Couplings $\lesssim$250 kHz

Owed to the fast T_M relaxation of the F_3Y_{122}• signal, contributions from F_3Y_{122}• are suppressed in measurements with τ = 620 ns. With such τ values, the ^{1}H background is an almost flat line that does not hamper the analysis of the ^{19}F spectra. Thus, no background correction was performed in these measurements.

Couplings of ~1.6 MHz

Obtaining background spectra. Irrelevant ^{1}H and ^{19}F contributions were removed by subtracting background, reference spectra. F_3Y_{122}-β_2/Y_{730}F-α_2 (95 µM, 42 s) was used to obtain spectra containing ^{1}H background signal of Y_{356}•, as this construct allows for high radical yields.[6] F_3Y_{122}-β_2 was used for the ^{19}F contributions from F_3Y_{122}•. Figure S9 shows the orientation selective ENDOR spectra of F_3Y_{122}-β_2/Y_{730}F-α_2 (purple lines) and F_3Y_{122}-β_2 (green lines). In the case of F_3Y_{122}-β_2/Y_{730}F-α_2, the most significant proton contribution is observed at the g_z position. At the g_x position, the ^{1}H resonances are weak and spread out over a larger area. For the ^{19}F background, the most significant contribution is observed at the g_x position, whereas the g_z position has essentially no background signal.

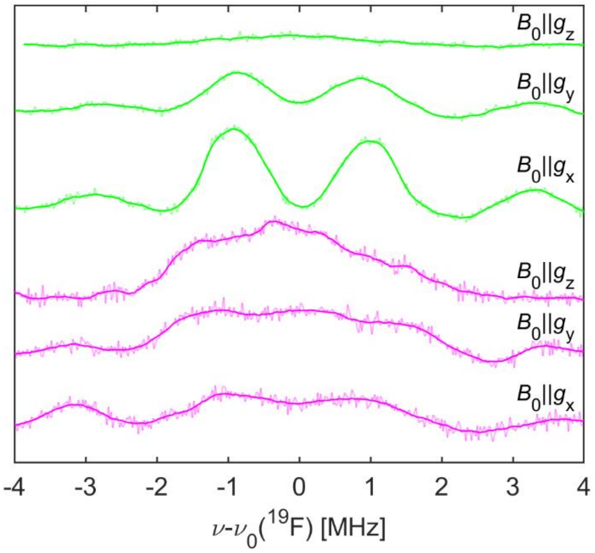

Figure S9. ENDOR spectra around the ^{19}F Larmor frequency recorded at 50 K of F_3Y_{122}-β_2/Y_{730}F-α_2 (purple lines, data have been smoothed, unsmoothed data are shown as partially transparent lines) and of F_3Y_{122}-β_2 (green lines, data have been smoothed, unsmoothed data are shown as partially transparent lines). All spectra are the sum of two measurements with τ values of 236 and 266 ns.

Background subtraction. The smoothed spectra shown in Figure S9 were subtracted from the ^{19}F ENDOR spectra obtained for the $E_{52}QF_3Y_{122}$-β_2/F_2Y_{731}-α_2 and F_3Y_{122}-β_2/F_2Y_{731}-α_2 samples. Characteristic features were used for scaling during the subtraction, with an estimated error of ±20% in scaling. The background subtraction started with the proton background of Y_{356}• (purple lines), where characteristic features at $B_0||g_z$ and $B_0||g_y$ were used for scaling. Then, the ^{19}F background from F_3Y_{122}• was subtracted (green lines), where features at the $B_0||g_x$ and $B_0||g_y$ were used. Figures S10 and S11 show the background correction on two representative ^{19}F ENDOR samples and the characteristic features used for scaling are indicated by arrows. The background corrected spectra are consistent with the spectra obtained at 80 K, where the contribution from F_3Y_{122}• is suppressed owed to fast relaxation (see below).

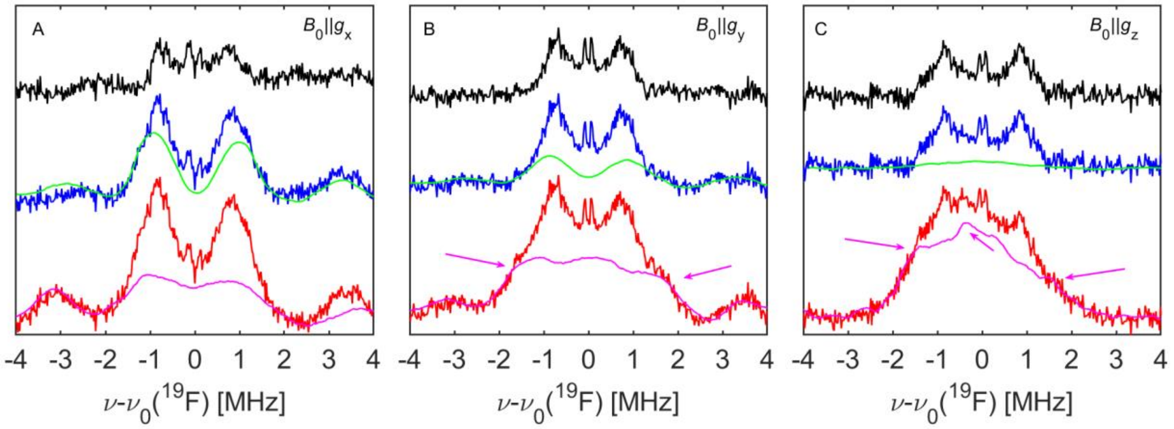

Figure S10. Background subtraction procedure for ^{19}F ENDOR spectra at 50 K of $E_{52}QF_3Y_{122}$-β_2/F_2Y_{731}-α_2 (80 µM, 35 s). Red lines show the spectra before background subtraction, blue lines after proton background subtraction, black lines after subtraction of proton and F_3Y_{122}• background. The background signals are scaled as they were used during subtraction and are the same as shown in Figure S9 using the same color code (purple lines F_3Y_{122}-β_2/$Y_{730}F$-α_2, green lines F_3Y_{122}-β_2). Characteristic features used for scaling the proton background from Y_{356}• are indicated by purple arrows. The other $E_{52}QF_3Y_{122}$-β_2/F_2Y_{731}-α_2 W-band sample with T_Q = 153 s yielded very similar results.

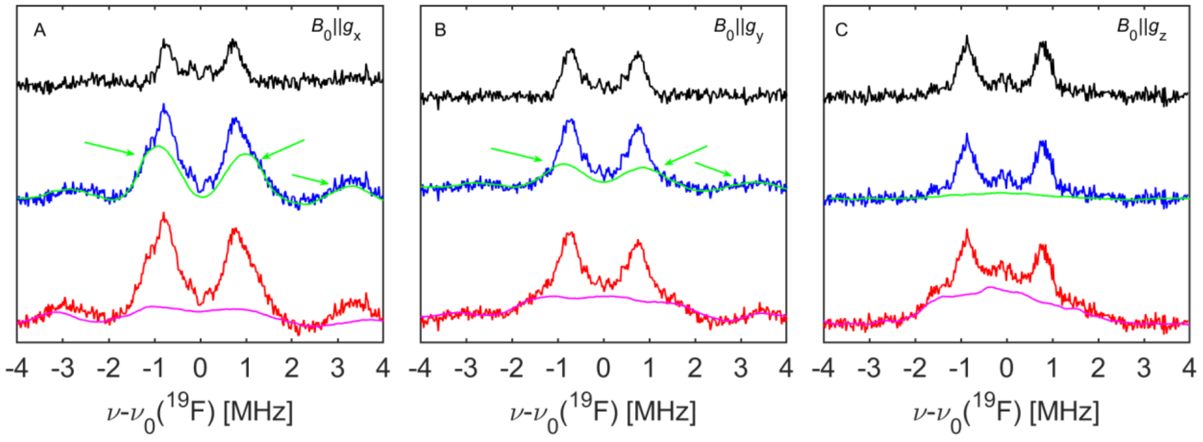

Figure S11. Background subtraction procedure for ^{19}F ENDOR spectra at 50 K of F_3Y_{122}-β_2/F_2Y_{731}-α_2 (80 µM, 50 s). Red lines show the spectra before background subtraction, blue lines after proton background subtraction, black lines after subtraction of proton and F_3Y_{122}• background. The background signals are scaled as they were used during subtraction and are the same as shown in Figure S9 using the same color code (purple lines F_3Y_{122}-β_2/$Y_{730}F$-α_2, green lines F_3Y_{122}-β_2). Characteristic features used for scaling the ^{19}F background from F_3Y_{122}• are indicated by green arrows. The other F_3Y_{122}-β_2/F_2Y_{731}-α_2 W-band sample with T_Q = 143 s yielded very similar results

5.5. EPR measurements at 80 K

W-band EPR and ENDOR measurements on Y_{356}• were performed at 80 K. The results show that the signal of unreacted F_3Y_{122}• is largely suppressed even for short τ values (~250 ns) owed to its fast relaxation. This is illustrated in Figure S12 by the echo detected EPR spectrum of F_3Y_{122}-β_2/F_2Y_{731}-α_2 (T_Q = 143 s) at 80 K, which has the lowest Y_{356}• yield and therefore the largest contribution of F_3Y_{122}•.

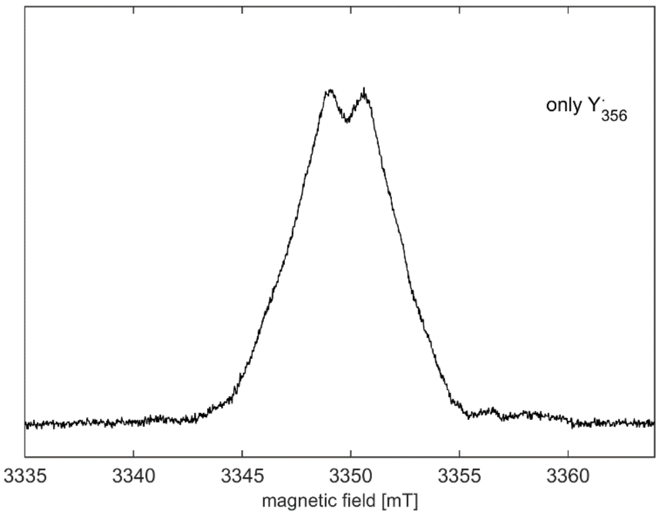

Figure S12. W-band echo detected EPR spectrum of F_3Y_{122}-β_2/F_2Y_{731}-α_2 (80 µM, T_Q =143 s) at 80 K. Experimental parameters: two-pulse echo sequence, π/2 pulse length = 12 ns, τ = 240 ns, shot-repetition-time = 2 ms, shots-per-point = 150, magnetic field axis resolution = 0.02 mT, 1 scan. A background signal stemming from the resonator and a signal the e' center[13] from the CFQ EPR tubes have been subtracted from the spectrum, contributing less than 10 % to the total signal intensity.

5.6. ^{19}F ENDOR at 80 K and comparison to 50 K

Most ENDOR measurements in this work were performed at 50 K to benefit from enhanced electron spin polarization. At 50 K the polarization is increased by 60% compared to 80 K, but the signal of F_3Y_{122}• is only suppressed for larger τ values ($\gtrsim$ 500 ns). This leads to the requirement of subtracting contributions from F_3Y_{122}• in the ^{19}F Mims ENDOR experiments with smaller τ values at T = 50 K. These contributions are suppressed at 80 K. Here, the results of ENDOR measurements at 80 K of two samples are presented and compared to results obtained at 50 K. At both temperatures, identical measurement parameters were used.

Figure S13 shows ENDOR spectra of $E_{52}QF_3Y_{122}$-β_2/F_2Y_{731}-α_2 (T_Q = 153 s) at 80 K (black lines) compared to spectra obtained at 50 K (red lines). For the 80 K measurements with short τ values (236 and 266 ns, Figure S13A), only ^{1}H resonances originating from Y_{356}• were subtracted, whereas it was also necessary to subtract ^{19}F resonances from unreacted F_3Y_{122}• in the 50 K measurements. Importantly, the spectra are indistinguishable at the two temperatures, which clearly shows that the background correction procedure to remove ^{19}F resonances from unreacted F_3Y_{122}• could be performed reliably for the measurements at 50 K.

Figure S13B shows measurements at 80 K with a larger τ value of 620 ns. Again, similar spectra are obtained at both temperatures, albeit the peak intensities are slightly different. This observation was attributed to relaxation effects. Noteworthy, the S/N in the spectra shown in Figure S13B at 80 K is worse by a factor of ~2.5 as compared to 50 K, despite similar measurement times (typically ~20 h per spectrum).

Similar results were obtained using another sample (F_3Y_{122}-β_2/F_2Y_{731}-α_2, 80 µM, 143 s), which is why all other measurements were subsequently performed at 50 K.

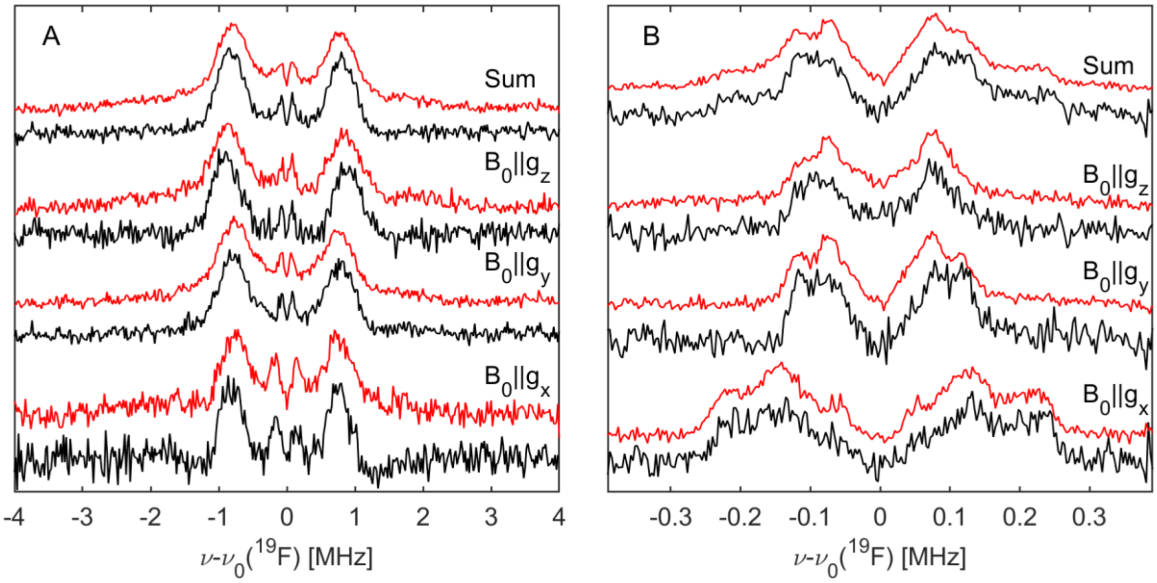

Figure S13. A) ^{19}F Mims ENDOR spectra of $E_{52}QF_3Y_{122}$-β_2/F_2Y_{731}-α_2 (~80 µM, 153 s) at 80 K (black lines, corrected for ^{1}H background of Y_{356}•) and 50 K (red lines, corrected ^{1}H background caused by Y_{356}• and ^{19}F background by F_3Y_{122}•) aimed at resolving the 1.6 MHz ^{19}F coupling. Measurement time per spectrum: ~10 h. B) ^{19}F Mims ENDOR spectra aimed at ^{19}F couplings < 500 kHz (τ = 620 ns) at 80 K (black) and 50 K (red). Measurement time per spectrum: ~20 h.

6. Control experiments with ^{17}O-Y-α_2

^{17}O-Y-α_2 was expressed to control whether any signs of a short distance between ^{17}O-Y_{731}(α) and Y_{356}•(β) could be obtained via ^{17}O ENDOR spectroscopy. Aside from the isotopic enrichment of the Y amino acids, ^{17}O-Y-α_2 is identical to the *wildtype* of the α subunit.

6.1. Preparation of ^{17}O-Y-α_2 and mass spectrometric determination of labelling degree

^{17}O-Y-labelled α_2 was expressed in analogy to previous protocols[14] by transforming the vector pET28-NrdA containing the ribonucleotide reductase α subunit (RNR α) gene into BL21 Star™ (DE3) One Shot™ Chemically Competent *E. coli* cells (Thermo Fisher Scientific Inc. Braunschweig, Germany) according to Inoue *et al*.[15] Single colonies were picked to inoculate a 200 mL starter culture containing 1x M9 salt solution (Sigma Aldrich),1 mM $MgSO_4$, 0.1 mM $CaCl_2$, 1% glycerol, 0.05% glucose, 0.2x MSM Trace Element solution,[16] 0.2 g/L of all 20 standard amino acids (except Tyr), 0.2 g/L of 35 – 40% ^{17}O enriched Tyrosine (Sigma Aldrich), and 50 µg/L kanamycin. It was grown at 37 °C, 200 rpm for 14 h. For the main culture, the same media was supplemented with 1 g/L glyphosate and inoculated to an optical density OD_{600} of 0.1. Gene expression was induced after growth to an OD_{600} of 0.6 at 37 °C by addition of 500 µM IPTG. The cells were harvested 4 h after induction by centrifugation, flash frozen in liquid nitrogen and stored at -80 °C. The purification proceeded in analogy to previous protocols,[14] yielding 22 mg purified protein per gram cell paste.

The activity of ^{17}O-Y-α_2 was assayed in a similar manner as described in section 1. The specific activity of ^{17}O-Y-α_2 was found to be identical to the specific activity of *wt*-α_2.

The labelling degree was confirmed by mass spectrometry (LC-MS/MS) on peptides obtained by digesting α_2 protein overnight by either trypsin or chymotrypsin. After C18 clean-up, the resulting peptides were subjected to LC-MS/MS analysis using an UltiMate 3000 RSLCnano HPLC system (Thermo Fisher Scientific) coupled online to a Q Exactive HF mass spectrometer (Thermo Fisher Scientific). We used the intensity ratio of the first two isotopic peaks to estimate the ^{17}O incorporation rate. This way, a labelling degree of 35 – 40% was obtained, in agreement with the labelling degree of the ^{17}O labeled tyrosine as specified by the manufacturer.

Figure S14 shows MS/MS data of three fragments of the peptide TL$Y_{730}Y_{731}$QNTR, which contains the interfacial Y residues of the α subunit, for the unlabeled *wt*-α_2 and for ^{17}O-Y-labelled α_2. The fragments labelled F_4, F_5, and F_6 contain either 0, 1, or 2 Y residues. The detected intensity ratio of the [m+1] to the [m] peak ($R_{det} = \frac{I([m+1])}{I([m])}$) increases significantly in the ^{17}O-Y labelled fragment spectra as compared to the unlabeled sample. Considering the amount of Y residues per fragment, a ^{17}O-Y incorporation of ~40% can be calculated, within error in agreement with the labelling degree of 35 – 40% of the ^{17}O-Y used during protein expression. Similar results were obtained for other peptides.

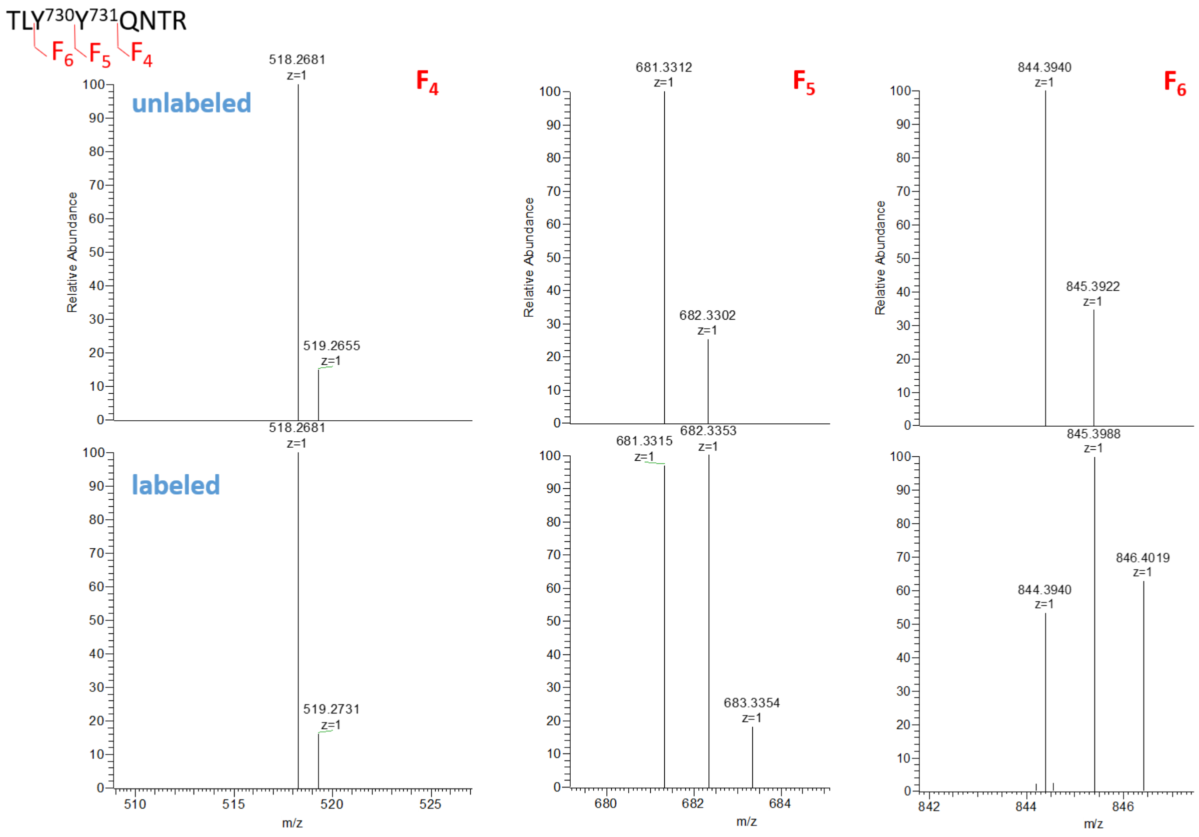

Figure S14. MS/MS data of three fragments of the peptide TLY$_{730}$Y$_{731}$QNTR. For unlabeled fragments F$_5$ and F$_6$, an intensity ratio of the [m+1]-peak compared to the [m]-peak of 35 and 45%, respectively, is calculated. The increased ratios in the labelled protein indicate ^{17}O-Y incorporation of ~40%.

6.2. ^{17}O ENDOR

^{17}O ENDOR measurements at 94 GHz/3.4 T were performed using parameters based on our recent ^{17}O ENDOR publication,[17] but adjustments to increase the measurement sensitivity were attempted (e.g. smaller RF window, longer τ values). Three different samples were investigated (Table S2), with radical yields of 15 – 20 % (F_3Y_{122}-β_2/^{17}O-Y-α_2 samples) or ca. 35% ($E_{52}QF_3Y_{122}$-β_2/^{17}O-Y-α_2). These yields agree with yields observed with F_2Y_{731}-α_2, confirming the functionality of ^{17}O-Y-α_2. Figure S15 shows one representative Mims ENDOR spectrum of each sample (grey lines, labelled **a** – **c**) around the ^{17}O Larmor frequency of ca. 19.35 MHz along with the data after 20-point smoothing (red lines). The figure also includes a similar ENDOR measurement conducted on the empty resonator (**d**) as well as a measurement on an $E_{52}QF_3Y_{122}$-β_2/*wt*-α_2 sample where the buffer contains 20 V% $^{17}OH_2$ (90% labelling degree, **e**). The data **a** – **c** are indistinguishable from the data obtained in the empty resonator (**d**), while data **e** show that even relatively small percentages of strongly coupled ^{17}O nuclei can be detected without problem. The effective concentration of Y_{356}•-^{17}O spin pairs in cases **c** and **e** is very similar and estimated as following. Neglecting incomplete loading of the diferric cofactor in β_2, one arrives at 4 – 5 µM for **c** (protein concentration · radical yield · percentage of trapped *flipped* conformer based on F_2Y_{731} experiments · labelling degree(Y_{731}) = 135 µM · 0.35 · 0.25 · 0.35 – 0.40) and at ca. 6 µM for the reference sample **e** (protein concentration · radical yield · V%($^{17}OH_2$) · labelling degree($^{17}OH_2$) = 100 µM · 0.35 · 0.2 · 0.9).

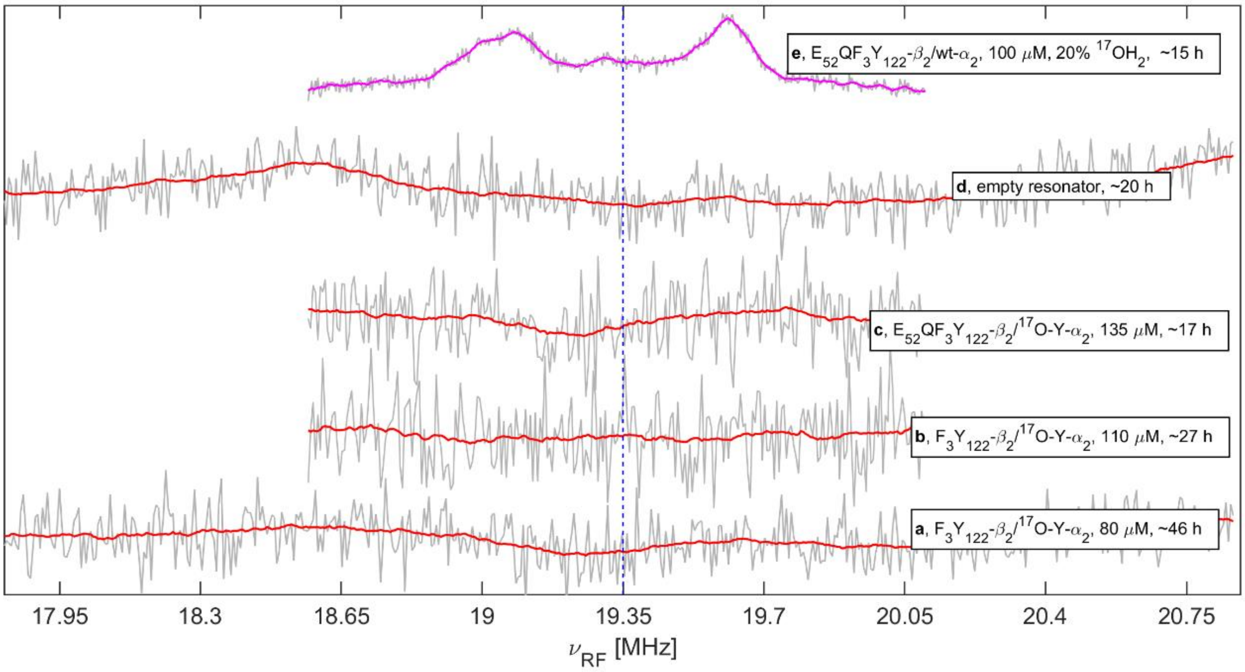

Figure S15. 94 GHz (W-band) Mims ENDOR spectra around the Larmor frequency of ^{17}O (blue dashes) on three different ^{17}O-Y-α_2 samples. Grey lines = experimental data, red and magenta lines = experimental data after 20 or 5 points data smoothing, respectively. The total measurement time is indicated. **a**: F_3Y_{122}-β_2/^{17}O-Y-α_2, 80 µM, radical yield 15 – 20 %, τ = 390 ns, 4500 scans, 512 points on RF axis, 6 kHz resolution. **b**: F_3Y_{122}-β_2/^{17}O-Y-α_2, 110 µM, radical yield 15 – 20 %, τ = 800 ns, 5200 scans, 256 points on RF axis, 6 kHz resolution. **c**: $E_{52}QF_3Y_{122}$-β_2/^{17}O-Y-α_2, 135 µM, radical yield ca. 35%, τ = 600 ns, 3300 scans, 256 points on RF axis, 6 kHz resolution. **d**: empty resonator, τ = 390 ns, 4500 scans, 512 points on RF axis, 6 kHz resolution. **e**: $E_{52}QF_3Y_{122}$-β_2/*wt*–α_2, 100 µM, radical yield ca. 35%, τ = 500 ns, 2200 scans, 512 points on RF axis, 3 kHz resolution. For all measurements, a shot-repetition-time of 2 ms with 20 shots per point was chosen and an RF pulse length of 40 µS was used.

7. DFT Calculations for the simulations of the ENDOR spectra

After extracting the tyrosine triad from the cryo-EM structure, geometry optimization was performed using the unrestricted Kohn-Sham method with the BP86 functional[18,19] and the def2-tzvp basis set.[20] For dispersion correction, Grimme's D3 correction with Becke-Johnson damping was used.[21–23] For the optimization, the non-hydrogen and non-fluorine atoms in F_2Y_{731} and Y_{730} were constrained in their XYZ coordinates. Furthermore, also the O – O distance between Y_{356}• and F_2Y_{731} was constrained. The geometry optimization of the triad aimed at correcting the covalent bond lengths to hydrogen and fluorine as well as the bond lengths in the tyrosyl radical to achieve a reasonable starting structure **S1** from which all other models could be derived in the subsequent modelling. These bond lengths could not be obtained correctly from the cryo-EM structure, as it did resolve neither the hydrogen atoms nor the location of tyrosyl radicals and also didn't contain fluorine labels at Y_{731}.

After the described geometry optimization, the HFC parameters were calculated using the B3LYP[19,24] functional with the def2-tzvpp basis set. Additionally, the RIJCOSX approximation was used (auxiliary basis set def2/J).[25,26] An error of ±20 % for the DFT derived EPR parameters is estimated. The CPCM(ethanol) keyword was used to include the assumed polarity of the radical's environment.

As mentioned in the main text, the presence of H-bond donors affects the radical's spin density distribution. H-bond donors reduce the spin density of the O atom of the phenoxyl radical,[27] which affects the coupling constants. In test calculations where we omitted the H-bonding water we could obtain similar coupling strengths as in the presented models **S4** and **S5** by increasing the O-O distance between F_2Y_{731} and Y_{356}• by 0.1 – 0.2 Å.

The calculated HFC parameters were used in the ENDOR simulations with only minor adjustments. First, the ORCA derived Euler angles and HFC constants were rounded to integer numbers (i.e. 1 kHz precision for the HFC constants). When adapting the Euler angles from the ORCA output-files, it was considered that EasySpin uses frame-rotation, while ORCA uses tensor-rotation. Therefore, the order of the Euler angles in the EasySpin input was reversed with respect to the ORCA output and the sign of each angle was changed, too.

Second, all ^{19}F HFC tensors were classified as to whether or not they were originating from purely dipolar hyperfine coupling. Purely dipolar interaction requires a vanishing isotropic coupling constant (a_{iso} = 0) and an anisotropic coupling tensor $\bar{\bar{T}}$ which is described by Equation (S6) (see also discussion in the main text):

$$\bar{\bar{T}} = T\begin{pmatrix} 2 & & \\ & -1 & \\ & & -1 \end{pmatrix} \qquad \text{(S6)}$$

Where the dipolar coupling constant T for a ^{19}F nucleus is given by Equation (S7):

$$T = \frac{74.52\ \text{MHz}\ \text{Å}^3}{R^3} \qquad \text{(S7)}$$

R is the distance between the centroid of the O, C1, C3, and C5 atoms of the phenoxyl radical and the ^{19}F nucleus, accounting for spin delocalization.[28] This dipolar, center-of-gravity approximation breaks down for short distances. Then, no equations like Equations (S6) and (S7) exists. Instead, the more general Equation (S8) has to be used:

$$\bar{\bar{A}} = \begin{pmatrix} T_x & & \\ & T_y & \\ & & T_z \end{pmatrix} + a_{iso} \ \ (\text{with } T_x + T_y + T_z = 0) \qquad \text{(S8)}$$

Nevertheless, the anisotropic coupling will in general increase when the distance between the unpaired electron and the ^{19}F nucleus decreases.

DFT usually predicts very small deviations from the purely dipolar case (Equations S6 and S7) which cannot be resolved experimentally.[11] Hence, all deviations from the purely dipolar case have been ignored in ENDOR simulations when the calculated isotropic coupling constant a_{iso} amounted to less than 10% of the anisotropic coupling constant T and was below 50 kHz. Likewise, deviations of the DFT derived anisotropic coupling constants from the tensor elements in Equation (S6) have been ignored when they did not exceed 10% of T. In cases of larger deviations, the HFC constants have been used without introducing any approximation (i.e. in accordance with Equation S8).

8. Models of Y_{356}•, F_2Y_{731}, and Y_{730}

Most models are shown in the main text, here we depict the remaining *stacked* model **S3** in Figure S16 and compare it to **S2** and, additionally, show the flipped Y dyad taken from the crystal structure of NH_2Y_{730}-α (no β subunit) in Figure S17.

To arrive at **S3**, the phenol plane of F_2Y_{731} in **S2** was rotated while monitoring how this affects the coupling of the ^{19}F nuclei. It was possible to obtain a structure **S3** in which couplings of ~250 kHz for the ^{19}F atom located closer to the radical are predicted by DFT. This value could potentially be assigned to F_b. Furthermore, the second, more weakly coupled ^{19}F nucleus in **S3** has a dipolar coupling constant of ~65 kHz that would fit F_d, but no explanation for the large coupling assigned to F_a is obtained.

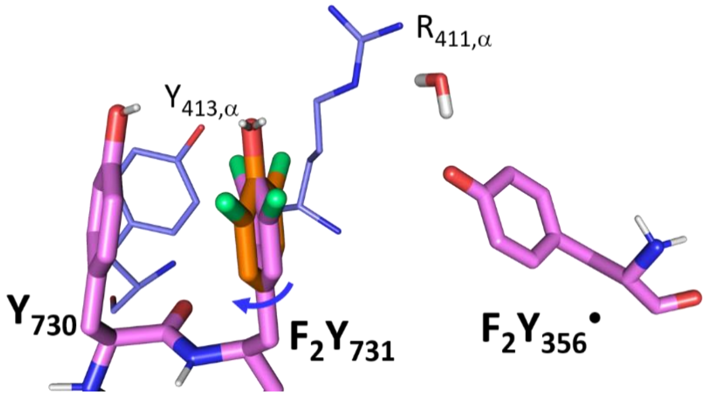

Figure S16. Comparison of **S2** (purple sticks) with **S3** (orange sticks). Since only F_2Y_{731} differs markedly between **S2** and **S3**, only this residue is shown for **S3**. The arrow indicates how **S3** was obtained from **S2** in PyMOL. Nearby R_{411} and Y_{413} (both α) are included as narrow sticks.

Figure S17 presents the implementation of the flipped Y-dyad from the crystal structure of NH_2Y_{730}-$\alpha 2$[14] (Bordeaux) into the cryo-EM structure and compares it to **S2** (semitransparent purple). The implementation was achieved by removing the NH_2 group from Y_{730} and adding ^{1}H and ^{19}F atoms where needed to the crystallographic dyad. As can be seen, flipped F_2Y_{731} directly derived from the crystal structure clashes with N_{733} (from the ordered $\alpha\beta$ pair of the cryo-EM structure).

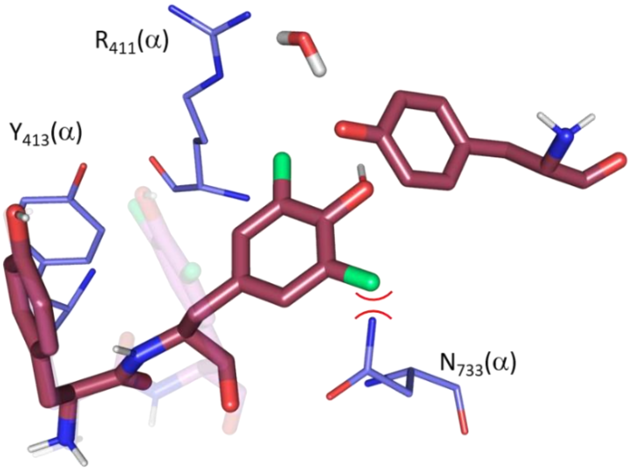

Figure S17. Comparison of the crystal structure derived Y-dyad Y_{730}-F_2Y_{731} (Bordeaux) to **S2** (semitransparent purple). Three nearby residues of the α subunit taken from the cryo-EM structure are included as thin, blue sticks. A clash with N_{733} is indicated.

Table S6 provides a summary description of each model structure and establishes hierarchical relations between the models.

Table S6. Qualitative description of models **S1** – **S5**, including the most significant geometrical modifications leading from one model to the next.

Model	Description
**S1**	Y triad from cryo-EM with the addition of F and H atoms where needed
**S2**	Based on **S1**, Y_{356}• repositioned and realigned by changing dihedral angles around Cα – N_{amino}, C_α – C_β, C_β – $C_{1,phenol}$. Centroid of Y_{356}• (based on O, C1, C3, and C5) was moved by ca 1 Å, distance between centroid of Y_{356}• and O of F_2Y_{731} reduced by ca. 0.5 Å.
**S3**	Based on **S2**, phenol plane of F_2Y_{731} turned by rotating around bond C_β – $C_{1,phenol}$ (ca 30°).
**S4**	Based on **S2**, F_2Y_{731} flipped towards Y_{356}• by rotating around C_α – C_β bond (ca. 70°). Phenol plane alignment of F_2Y_{731} was changed by rotating around C_β – $C_{1,phenol}$ (ca. 30°).
**S5**	Based on **S4**. F_2Y_{731} repositioned and realigned by changing dihedral angles around Cα – N_{amino}, C_α – C_β, C_β – $C_{1,phenol}$ (ca. 10, 10, and 60°, respectively) to align the dipolar vector and the C-O• bond of Y_{356}•.

Key geometrical parameters of the final model structures **S1** – **S5** are given in Table S7. The xyz coordinates of each model can be found in Section SI10. We note that many more models were tested, but to keep the amount of text manageable we only include the most significant ones herein.

Table S7. Key geometrical parameters of the different models discussed in the text.

	F•-Y_{356}[a] [Å]	O_{Y731}-O_{Y356} [Å]	Y_{356}•-F_3Y_{122}•[a,b] [Å]	F_2Y_{731}-F_3Y_{122}•[a,b] [Å]	Figure[c]
**S1**	8.9/10.5	8.3	30.1	38.2	-
**S2**	8.4/9.8	7.4	30.5	38.2	5 + 8,
**S3**	7.3/10.7	7.4	30.5	38.2	S15
**S4**	4.1/6.8	3.1	30.5	35.0	7
**S5**	4.4/7.3	3.0	30.5	35.5	7 + 8

[a] Measured using the centroid of the O, C1, C3 and C5 atoms of the tyrosines as point of reference. [b] Distance measured between F_3Y_{122} in α'β' (β-tail disordered) and Y_{356} in αβ (β-tail ordered). [c] This column gives the numbers of the Figures in which the model structure or simulations based on the respective model are shown.

Table S8 lists all DFT derived ^{19}F HFC parameters for models **S1** – **S5**.

Table S8. DFT derived ^{19}F HFC parameters used in the simulations of the different models.

**Structure**	**F Atom**[a]	T_x, T_y, T_z[b] **[kHz]**	a_{iso} **[kHz]**	α, β, γ[c] **[°]**
**S1**	d	-63, -66, 129	0	47, -61, 25
	c	-112, 228, -116	0	-79, -6, 30
**S2**	d	-86, 169, -83	0	-47, -71, -16
	c	-151, -154, 306	0	22, -79, 26
**S3**	d	-65, 132, -67	0	-51, -76, -10
	b	-243, -246, 486	-1	23, -83, 19
**S4**	b	-181, 508, -327	-4	-71, -43, 36
	a	-1072, 1930, -858	-757	-11, -85, 4
**S5**	b	-242, -250, 491	-16	19, -78, -27
	a	1593, -654, -939	-1013	83, -12, 22

[a] Possible assignment based on size of HFC constant and orientation selectivity behavior. [b] Order as given in ORCA output-files after redefining the orientation of the *g* tensor ($g_{max} = g_x$, $g_{min} = g_z$). [c] Values as used for EasySpin input.

Finally, models **S1** – **S5** were also used to calculate the expected coupling constants of the phenol O-atom of Y_{731}. These values should allow estimating coupling constants of *stacked* and *flipped* conformers in ^{17}O-Y-α_2 samples (Table S9).

Table S9. DFT derived ^{17}O HFC parameters.

Structure	T_x, T_y, T_z [kHz]	a_{iso} [kHz]	A_x, A_y, A_z[a] [kHz]
**S1**	-12, 6, 6	0	-12, 6, 6
**S2**	-31, 16, 16	0	-14, 7, 7
**S3**	-31, 16, 15	0	-31, 15, 15
**S4**	269, -4, -265	551	820, 548, 286
**S5**	166, -15, -151	328	494, 313, 177

[a] $A_i = T_i + a_{iso}$

9. Optimizing the stacked/flipped ratio in simulations of ENDOR spectra for different samples

The different ENDOR samples (different mutants and quenching times) showed slight variations in their *stacked/flipped* ratios. The root-mean square deviation (*rmsd*) between simulated (based on structures **S2** and **S5**) and experimental data was calculated for the sum spectra of both short (236/266 ns) and long (~620 ns) τ value measurements in dependence of the ratio of *stacked* and *flipped* conformations to find the optimal value. The *rmsd* was calculated using equation (S9), where *I* represents the intensity of either simulated or experimental data and *N* the number of data points:

$$rmsd = \sqrt{\frac{\sum(I_{sim} - I_{exp})^2}{N}} \quad \text{(S9)}$$

Figure S18 shows the results of these calculations for each sample in dependence of the percentage of flipped conformer and, equivalently, the ratio *stacked/flipped*.

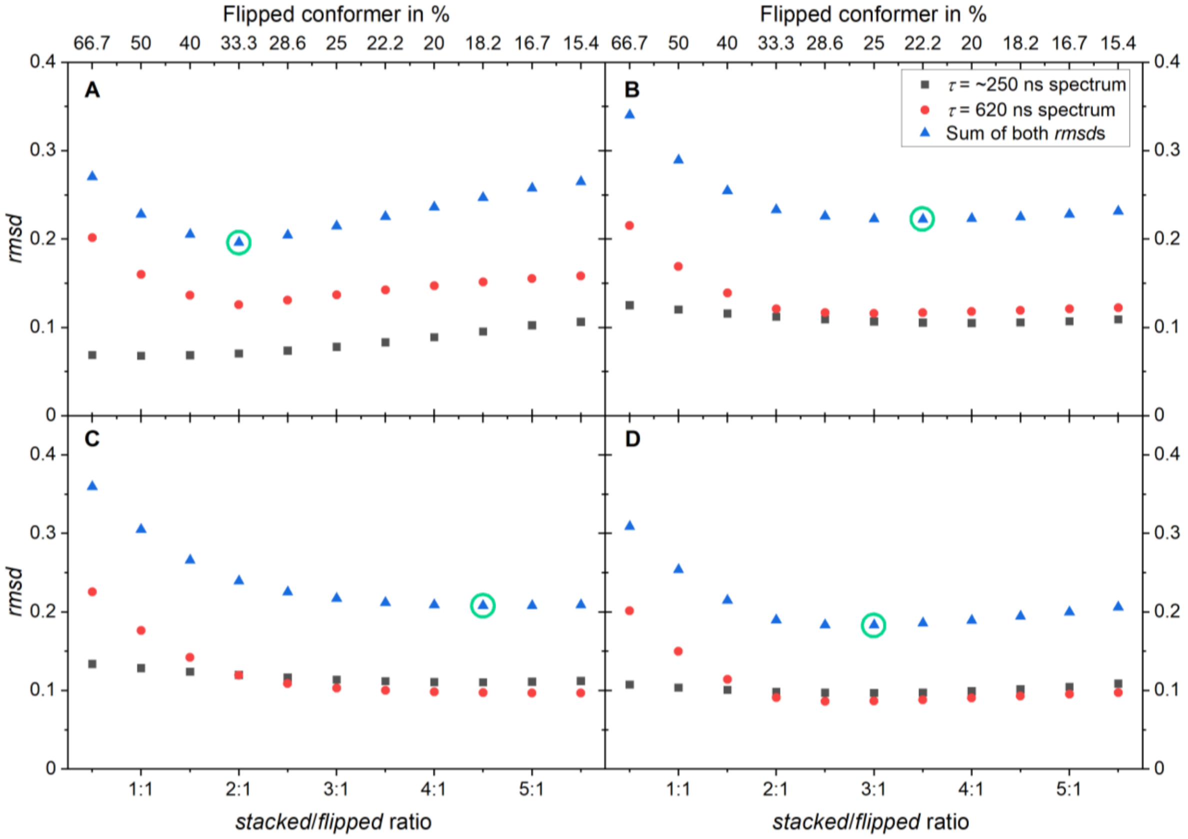

Figure S18. *rmsd* of the sum ENDOR spectra in dependence of the *stacked/flipped* ratio of F_3Y_{122}-β_2/F_2Y_{731}-α_2, 83 µM, T_Q = 50 s (**A**), F_3Y_{122}-β_2/F_2Y_{731}-α_2, 83 µM, T_Q = 143 s (**B**), $E_{52}QF_3Y_{122}$-β_2/F_2Y_{731}-α_2, 83 µM, T_Q = 35 s (**C**), and $E_{52}QF_3Y_{122}$-β_2/F_2Y_{731}-α_2, 83 µM, T_Q = 153 s (**D**). The minima/optimal ratios are indicated by green circles.

10. References

(1) Lin, Q.; Parker, M. J.; Taguchi, A. T.; Ravichandran, K.; Kim, A.; Kang, G.; Shao, J.; Drennan, C. L.; Stubbe, J. Glutamate 52-β at the α/β Subunit Interface of Escherichia Coli Class Ia Ribonucleotide Reductase Is Essential for Conformational Gating of Radical Transfer. *J. Biol. Chem.* **2017**, *292* (22), 9229–9239.

(2) Ge, J.; Yu, G.; Ator, M. A.; Stubbe, J. Pre-Steady-State and Steady-State Kinetic Analysis of E. Coli Class I Ribonucleotide Reductase. *Biochemistry* **2003**, *42* (34), 10071–10083.

(3) Yokoyama, K.; Smith, A. A.; Corzilius, B.; Griffin, R. G.; Stubbe, J. Equilibration of Tyrosyl Radicals (Y356•, Y731•, Y730•) in the Radical Propagation Pathway of the Escherichia Coli Class Ia Ribonucleotide Reductase. *J. Am. Chem. Soc.* **2011**, *133* (45), 18420–18432.

(4) Minnihan, E. C.; Young, D. D.; Schultz, P. G.; Stubbe, J. Incorporation of Fluorotyrosines into Ribonucleotide Reductase Using an Evolved, Polyspecific Aminoacyl-TRNA Synthetase. *J. Am. Chem. Soc.* **2011**, *133* (40), 15942–15945.

(5) Oyala, P. H.; Ravichandran, K. R.; Funk, M. A.; Stucky, P. A.; Stich, T. A.; Drennan, C. L.; Britt, R. D.; Stubbe, J. Biophysical Characterization of Fluorotyrosine Probes Site-Specifically Incorporated into Enzymes: E. Coli Ribonucleotide Reductase As an Example. *J. Am. Chem. Soc.* **2016**, *138* (25), 7951–7964.

(6) Nick, T. U.; Ravichandran, K. R.; Stubbe, J.; Kasanmascheff, M.; Bennati, M. Spectroscopic Evidence for a H Bond Network at Y356 Located at the Subunit Interface of Active E. Coli Ribonucleotide Reductase. *Biochemistry* **2017**, *56* (28), 3647–3656.

(7) Kasanmascheff, M.; Lee, W.; Nick, T. U.; Stubbe, J.; Bennati, M. Radical Transfer in E. Coli Ribonucleotide Reductase: A NH 2 Y 731/R 411 A-α Mutant Unmasks a New Conformation of the Pathway Residue 731. *Chem. Sci.* **2016**, *7* (3), 2170–2178.

(8) Ravichandran, K. R.; Taguchi, A. T.; Wei, Y.; Tommos, C.; Nocera, D. G.; Stubbe, J. A> 200 MeV Uphill Thermodynamic Landscape for Radical Transport in Escherichia Coli Ribonucleotide Reductase Determined Using Fluorotyrosine-Substituted Enzymes. *J. Am. Chem. Soc.* **2016**, *138* (41), 13706–13716.

(9) Chiang, Y.-W.; Borbat, P. P.; Freed, J. H. The Determination of Pair Distance Distributions by Pulsed ESR Using Tikhonov Regularization. *J. Magn. Reson.* **2005**, *172* (2), 279–295.

(10) Jeschke, G.; Chechik, V.; Ionita, P.; Godt, A.; Zimmermann, H.; Banham, J.; Timmel, C.; Hilger, D.; Jung, H. DeerAnalysis2006—a Comprehensive Software Package for Analyzing Pulsed ELDOR Data. *Appl. Magn. Reson.* **2006**, *30* (3–4), 473–498.

(11) Meyer, A.; Dechert, S.; Dey, S.; Höbartner, C.; Bennati, M. Measurement of Angstrom to Nanometer Molecular Distances with 19F Nuclear Spins by EPR/ENDOR Spectroscopy. *Angew. Chem. Int. Ed.* **2020**, *59* (1), 373–379.

(12) Tkach, I.; Bejenke, I.; Hecker, F.; Kehl, A.; Kasanmascheff, M.; Gromov, I.; Prisecaru, I.; Höfer, P.; Hiller, M.; Bennati, M. 1H High Field Electron-Nuclear Double Resonance Spectroscopy at 263 GHz/9.4 T. *J. Magn. Reson.* **2019**, *303*, 17–27.

(13) Weeks, R.; Nelson, C. Trapped Electrons in Irradiated Quartz and Silica: II, Electron Spin Resonance. *J. Am. Ceram. Soc.* **1960**, *43* (8), 399–404.

(14) Minnihan, E. C.; Seyedsayamdost, M. R.; Uhlin, U.; Stubbe, J. Kinetics of Radical Intermediate Formation and Deoxynucleotide Production in 3-Aminotyrosine-Substituted Escherichia Coli Ribonucleotide Reductases. *J. Am. Chem. Soc.* **2011**, *133* (24), 9430–9440.
(15) Inoue, H.; Nojima, H.; Okayama, H. High Efficiency Transformation of Escherichia Coli with Plasmids. *Gene* **1990**, *96* (1), 23–28.
(16) Christel, S.; Dopson, M. MSM Trace Element (TE) Solution. **2015**.
(17) Hecker, F.; Stubbe, J.; Bennati, M. Detection of Water Molecules on the Radical Transfer Pathway of Ribonucleotide Reductase by 17O Electron–Nuclear Double Resonance Spectroscopy. *J. Am. Chem. Soc.* **2021**, *143* (19), 7237–7241.
(18) Perdew, J. P. Density-Functional Approximation for the Correlation Energy of the Inhomogeneous Electron Gas. *Phys. Rev. B* **1986**, *33* (12), 8822.
(19) Becke, A. D. Density-Functional Exchange-Energy Approximation with Correct Asymptotic Behavior. *Phys. Rev. A* **1988**, *38* (6), 3098.
(20) Weigend, F.; Ahlrichs, R. Balanced Basis Sets of Split Valence, Triple Zeta Valence and Quadruple Zeta Valence Quality for H to Rn: Design and Assessment of Accuracy. *Phys. Chem. Chem. Phys.* **2005**, *7* (18), 3297–3305.
(21) Grimme, S.; Antony, J.; Ehrlich, S.; Krieg, H. A Consistent and Accurate Ab Initio Parametrization of Density Functional Dispersion Correction (DFT-D) for the 94 Elements H-Pu. *J. Chem. Phys.* **2010**, *132* (15), 154104.
(22) Grimme, S.; Ehrlich, S.; Goerigk, L. Effect of the Damping Function in Dispersion Corrected Density Functional Theory. *J. Comput. Chem.* **2011**, *32* (7), 1456–1465.
(23) Becke, A. D.; Johnson, E. R. A Density-Functional Model of the Dispersion Interaction. *J. Chem. Phys.* **2005**, *123* (15), 154101.
(24) Lee, C.; Yang, W.; Parr, R. G. Development of the Colle-Salvetti Correlation-Energy Formula into a Functional of the Electron Density. *Phys. Rev. B* **1988**, *37* (2), 785.
(25) Neese, F. An Improvement of the Resolution of the Identity Approximation for the Formation of the Coulomb Matrix. *J. Comput. Chem.* **2003**, *24* (14), 1740–1747.
(26) Neese, F.; Wennmohs, F.; Hansen, A.; Becker, U. Efficient, Approximate and Parallel Hartree–Fock and Hybrid DFT Calculations. A 'Chain-of-Spheres' Algorithm for the Hartree–Fock Exchange. *Chem. Phys.* **2009**, *356* (1–3), 98–109.
(27) Lucarini, M.; Mugnaini, V.; Pedulli, G. F.; Guerra, M. Hydrogen-Bonding Effects on the Properties of Phenoxyl Radicals. An EPR, Kinetic, and Computational Study. *J. Am. Chem. Soc.* **2003**, *125* (27), 8318–8329.
(28) Riplinger, C.; Kao, J. P.; Rosen, G. M.; Kathirvelu, V.; Eaton, G. R.; Eaton, S. S.; Kutateladze, A.; Neese, F. Interaction of Radical Pairs Through-Bond and through-Space: Scope and Limitations of the Point− Dipole Approximation in Electron Paramagnetic Resonance Spectroscopy. *J. Am. Chem. Soc.* **2009**, *131* (29), 10092–10106.

Appendix. XYZ coordinates of models S2 and S5

S2

N	128.287231000	132.835419000	108.464737000
C	129.719208000	132.803329000	108.352562000
C	130.163071000	131.628525000	107.503021000
O	129.446167000	130.690384000	107.225273000
C	130.528488000	132.668747000	109.707062000
C	130.420151000	133.916534000	110.516357000
C	130.678131000	135.180344000	109.926521000
C	129.984619000	133.869888000	111.862755000
C	130.520798000	136.351807000	110.628563000
C	129.825272000	135.025269000	112.593925000
C	130.090851000	136.335068000	112.014565000
O	129.935974000	137.370834000	112.726837000
H	127.894836000	131.894440000	108.401283000
H	130.092453000	133.722305000	107.861710000
H	131.233734000	131.660782000	107.180412000
H	131.583084000	132.464828000	109.457222000
H	130.128433000	131.805954000	110.259216000
H	131.008423000	135.216751000	108.885834000
H	129.786438000	132.898117000	112.319939000
H	130.709305000	137.313065000	110.158600000
H	129.498367000	135.007385000	113.633949000
H	127.958588000	133.309036000	109.304329000
N	137.499268000	146.460266000	111.518837000
C	136.304901000	146.086670000	112.286690000
C	135.744522000	144.711472000	111.923058000
O	135.851517000	143.766190000	112.705666000
C	135.244827000	147.174377000	112.102615000
C	134.083755000	147.096817000	113.056328000
C	134.248581000	146.616791000	114.340881000
C	132.817032000	147.502869000	112.665657000
C	133.187729000	146.544128000	115.212868000
C	131.751144000	147.441238000	113.529701000
C	131.940002000	146.955383000	114.803268000
O	130.878159000	146.880722000	115.674240000
H	138.234894000	145.761536000	111.643059000
H	137.860184000	147.347565000	111.873360000
H	136.530426000	145.979309000	113.359550000
H	134.885773000	147.177856000	111.061646000
H	135.754150000	148.149323000	112.228996000
H	135.220459000	146.269150000	114.686966000
H	132.664917000	147.869370000	111.647522000
H	133.309784000	146.143478000	116.219315000
H	130.757797000	147.763672000	113.208046000
H	130.054840000	146.906601000	115.156456000
N	135.182068000	144.567291000	110.723793000
C	134.455017000	143.349365000	110.368698000
C	135.232040000	142.492508000	109.376434000
O	136.327713000	142.837799000	108.928177000

C	133.075058000	143.674042000	109.796257000
C	132.003387000	143.944443000	110.819283000
C	132.174622000	143.584198000	112.143562000
C	130.803833000	144.533661000	110.450867000
C	131.187317000	143.821503000	113.077591000
C	129.814529000	144.776001000	111.374924000
C	130.011765000	144.419952000	112.687294000
O	129.029465000	144.659286000	113.616348000
H	134.395660000	142.747559000	111.289444000
H	134.746948000	141.530289000	109.080048000
H	132.749130000	142.827133000	109.163284000
H	133.168411000	144.528412000	109.106178000
H	133.100815000	143.150803000	112.520073000
H	130.631302000	144.830124000	109.413193000
F	131.357498000	143.487610000	114.369690000
F	128.632568000	145.363770000	111.037888000
H	128.247101000	144.987518000	113.135208000
H	135.240143000	145.321243000	110.045998000
O	128.388367000	137.475632000	115.078667000
H	129.103821000	137.356262000	115.724548000
H	128.861694000	137.436188000	114.218872000

S5

N	128.287231000	132.835419000	108.464737000
C	129.719208000	132.803329000	108.352562000
C	130.163071000	131.628525000	107.503021000
O	129.446167000	130.690384000	107.225273000
C	130.528488000	132.668747000	109.707062000
C	130.420151000	133.916534000	110.516357000
C	130.678131000	135.180344000	109.926521000
C	129.984619000	133.869888000	111.862755000
C	130.520798000	136.351807000	110.628563000
C	129.825272000	135.025269000	112.593925000
C	130.090851000	136.335068000	112.014565000
O	129.935974000	137.370834000	112.726837000
H	127.894836000	131.894440000	108.401283000
H	130.092453000	133.722305000	107.861710000
H	131.233734000	131.660782000	107.180412000
H	131.583084000	132.464828000	109.457222000
H	130.128433000	131.805954000	110.259216000
H	131.008423000	135.216751000	108.885834000
H	129.786438000	132.898117000	112.319939000
H	130.709305000	137.313065000	110.158600000
H	129.498367000	135.007385000	113.633949000
H	127.958588000	133.309036000	109.304329000
N	137.516556000	146.528015000	111.649582000
C	136.311081000	146.131241000	112.387878000
C	135.760696000	144.764725000	111.979088000
O	135.857864000	143.799377000	112.738121000
C	135.250885000	147.220749000	112.215439000

C	134.074844000	147.115082000	113.147858000
C	134.220367000	146.601715000	114.421806000
C	132.813385000	147.528122000	112.747581000
C	133.145844000	146.503357000	115.274292000
C	131.733902000	147.440979000	113.592346000
C	131.903656000	146.922134000	114.855545000
O	130.828140000	146.821793000	115.706947000
H	138.255814000	145.833664000	111.776009000
H	137.863159000	147.413147000	112.023170000
H	136.520554000	145.996475000	113.461098000
H	134.909164000	147.248596000	111.169312000
H	135.754379000	148.193787000	112.376503000
H	135.188431000	146.251205000	114.775299000
H	132.679352000	147.930084000	111.740067000
H	133.257324000	146.088593000	116.276474000
H	130.745407000	147.772476000	113.262268000
H	130.039902000	147.152481000	115.243210000
N	135.218063000	144.650848000	110.767494000
C	134.500427000	143.440887000	110.368690000
C	135.053146000	142.852081000	109.076317000
O	135.977753000	143.382416000	108.456299000
C	133.003799000	143.709991000	110.210983000
C	132.100449000	142.803955000	111.007507000
C	131.958282000	142.962143000	112.374039000
C	131.414124000	141.768951000	110.393341000
C	131.140686000	142.125839000	113.104759000
C	130.594589000	140.930084000	111.112244000
C	130.460129000	141.113388000	112.467453000
O	129.643524000	140.281723000	113.192688000
H	134.675888000	142.719086000	111.185471000
H	134.561340000	141.942383000	108.670341000
H	132.725281000	143.670090000	109.142395000
H	132.828339000	144.747025000	110.531517000
H	132.439240000	143.765594000	112.924446000
H	131.493683000	141.617981000	109.314728000
F	130.989243000	142.318192000	114.435112000
F	129.907684000	139.944870000	110.502884000
H	129.869019000	139.338181000	113.007813000
H	135.355423000	145.392731000	110.086601000
O	128.388367000	137.475632000	115.078667000
H	129.103821000	137.356262000	115.724548000
H	128.861694000	137.436188000	114.218872000
